# The nexus between reactive oxygen species and the mechanism of action of herbicides

**DOI:** 10.1016/j.jbc.2023.105267

**Published:** 2023-09-19

**Authors:** Catherine Traxler, Todd A. Gaines, Anita Küpper, Peter Luemmen, Franck E. Dayan

**Affiliations:** 1 Department of Agricultural Biology, Colorado State University, Fort Collins, Colorado, USA; 2 Plant Biotechnology Division, Bayer CropScience, Chesterfield, Missouri, USA; 3 Research & Development Division, Bayer AG, Industriepark Höchst, Frankfurt am Main, Germany

**Keywords:** reactive oxygen species, antioxidant, hydrogen peroxide, lipid peroxidation, oxidative stress, superoxide ion, oxygen radicals, plant physiology, plant biochemistry

## Abstract

Herbicides are small molecules that act by inhibiting specific molecular target sites within primary plant metabolic pathways resulting in catastrophic and lethal consequences. The stress induced by herbicides generates reactive oxygen species (ROS), but little is known about the nexus between each herbicide mode of action (MoA) and their respective ability to induce ROS formation. Indeed, some herbicides cause dramatic surges in ROS levels as part of their primary MoA, whereas other herbicides may generate some ROS as a secondary effect of the stress they imposed on plants. In this review, we discuss the types of ROS and their respective reactivity and describe their involvement for each known MoA based on the new Herbicide Resistance Action Committee classification.

Molecular oxygen (O_2_) was introduced to the earth’s atmosphere as a byproduct released by photosynthetic organisms about 2.7 billion years ago (1). This seemingly innocuous waste product now accounts for nearly 21% of the atmosphere. Molecular oxygen primarily exists in its diatomic form O_2_ and is most stable in its triplet state (triplet oxygen, ^3^O_2_), as atomic energy is very reactive. While aerobic organisms rely on oxygen as a terminal electron acceptor sustaining respiration, O_2_ is a reactive molecule that is readily converted into many forms of reactive oxygen species (ROS) (1). ROS is a collective term that includes both oxygen radicals and some nonradicals that are easily converted into radicals or serve as oxidizing agents. Not all ROS have the same level of reactivity. For example, hydrogen peroxide (H_2_O_2_) and superoxide (O_2_^•−^) react fast with few molecules, whereas the hydroxyl radical (^•^OH) reacts quickly with many molecules, and other ROS may have intermediate reactivities (Table 1 and Fig. 1*A*).

**Table 1:** Different members of the ROS family and their attributes

ROS	*T*_1/2_ (μs)	Subcellular origins	Mechanism of action	Reaction with DNA	Reaction with protein
Superoxide (O_2_^•−^)	1–4	Membranes, chloroplasts, and mitochondria	Reacts with redox-active proteins (*e.g.*, Fe–S proteins)	None or extremely low	*Via* the Fe center
Hydroxyl radical (^•^OH)	<1	Membranes, chloroplasts, and mitochondria	Extremely reactive with all biomolecules	Very reactive	Very reactive
H_2_O_2_	1000	Membranes, chloroplasts, mitochondria, and peroxisome	Oxidizes proteins and forms ^•^OH *via* O_2_^•−^	None or extremely low	Attacks Cys residue
Singlet oxygen (^1^O_2_)	1–4	Membranes, chloroplasts, and mitochondria	Oxidizes proteins, polyunsaturated fatty acids, and DNA	Very reactive	Attacks Trp, His, Tyr, Met, and Cys residues

**Figure 1:**
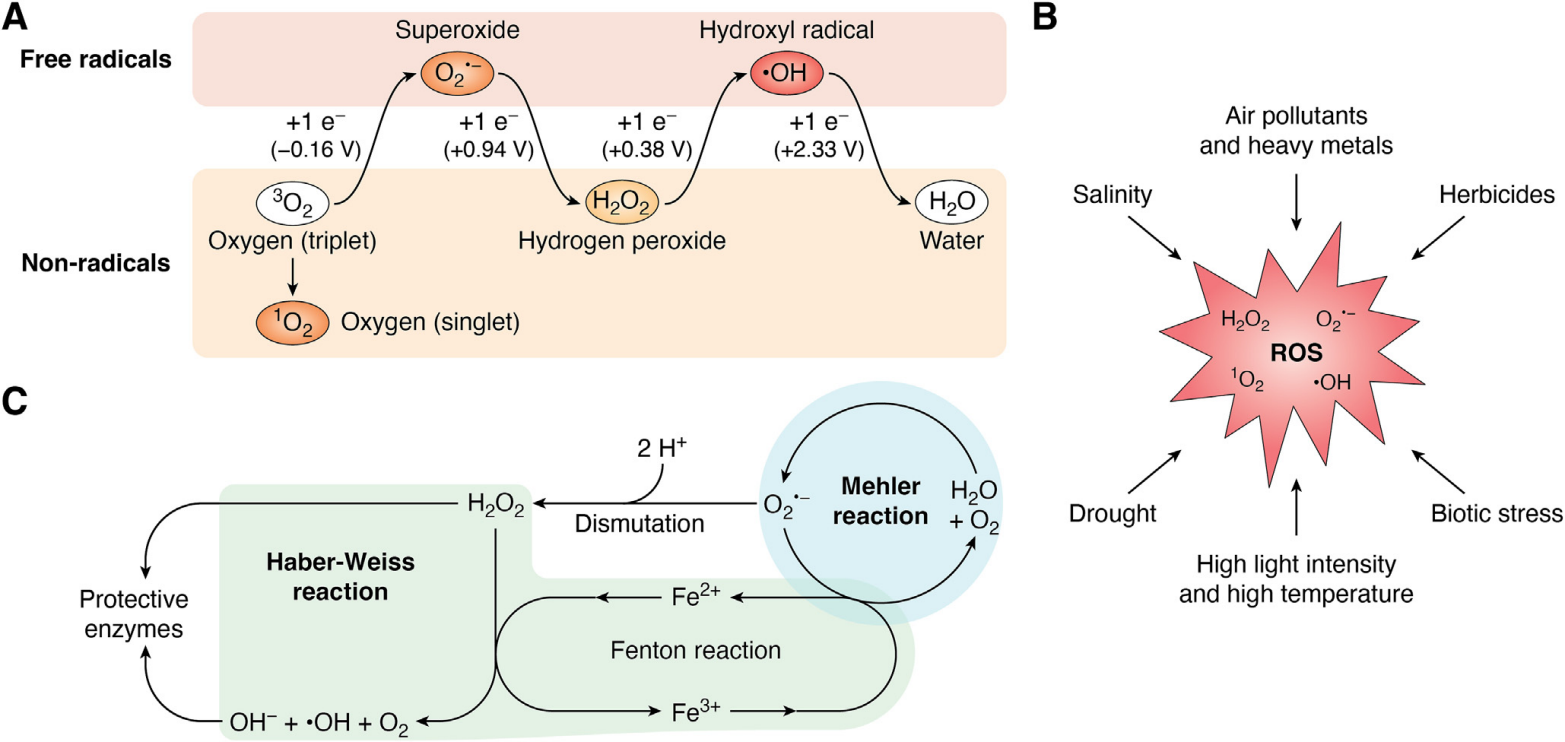
**Overview of reactive oxygen species in plants.***A*, main ROS in plants, separated whether they exist as nonradicals or free radicals. Color indicates their overall reactivity, *pale orange* = low reactivity, *orange* = intermediate reactivity, and *red* = highly reactive. Reduction potentials for oxygen species shown in parentheses are from the study by Imlay (183). *B*, factors involved in the generation of ROS in plants. *C*, summary of the relationship between the Mehler, Haber–Weiss, and Fenton reactions. The Mehler reaction oxidizes water and oxygen to O_2_^•−^, which is subsequently dismutated into H_2_O_2_ by superoxide dismutase (SOD). The Haber–Weiss and Fenton reactions oxidize H_2_O_2_ to highly reactive ^•^OH and OH^−^ in plants (see text for more detailed description of these reactions). H_2_O_2_, hydrogen peroxide; O_2_^•−^, superoxide; ^•^OH, hydroxyl radical; OH^−^, hydroxide; ROS, reactive oxygen species.

While plants (and most other organisms) generate basal levels of ROS through normal physiological processes within various cellular compartments (*e.g.*, chloroplasts, mitochondria, and peroxisomes) (Table 1), these ROS do not accumulate to lethal levels because they are scavenged by a robust network of antioxidant mechanisms (2). Under normal conditions, less than 2% of the O_2_ consumption by plant tissues results in ROS production. However, the delicate balance between the production of ROS and ROS scavenging mechanisms can be disrupted by different types of stresses (Fig. 1*B*). Consequently, the well-being of plants depends on many important factors like change in growth conditions, severity and duration of stress conditions, and the capacity of the plants to quickly adapt to bursts of ROS accumulation (3).

ROS can be produced by cells as an indicator of stress. Though commonly thought of as just a sign of stress and agents of damage in plants, a new prevailing opinion suggests that ROS are more than just destructive, playing vital roles in signaling and adaptation in the plant (4). This role of signaling and adaptation is involved in the control of programmed cell death, defense against pathogens, and stomatal conductance (5). This hypothesis suggests that ROS-related damage that eventually leads to cell death is not accidental but instead occurs because of activation of signaling pathways that lead to cell death (6). Despite the aspects of their hypothesized role in signaling and adaptation, ROS can still function as extremely phytotoxic agents, causing oxidative damage to proteins, DNA, and lipids (7).

Unpaired electrons from ROS can be donated to other molecules, or the ROS can instead take away an electron from another molecule to increase its own stability. These reactions form new radicals driving a chain reaction generating more radicals, the process only ending when two radicals react with each other (8). This chain reaction is most evident in the process of lipid peroxidation where a radical will react with a membrane lipid, forming a lipid radical, that subsequently react with neighboring lipids, forming lipid peroxides, hydroperoxides, and alkyl chain radicals altering the fluidity and disrupting the integrity of the membrane (9). Ultimately, ROS accumulation can lead to cell component damage, and lipid peroxidation can erupt leading to membrane breakdown and eventual cell death.

The most abundant ROS produced during cellular processes is H_2_O_2_, which is the most stable and likely candidate for stress signaling because of its ability to diffuse throughout the cell (10, 11). Regulated increases in H_2_O_2_ concentration in plants induce reversible oxidation and lead to alterations in protein structure or enzymatic function, which is the basis for how H_2_O_2_ mediates signaling. When these concentrations exceed that of this regulated increase, H_2_O_2_ can cause irreversible oxidative damage to various molecules (12). H_2_O_2_ targets bound and unbound iron, damaging iron–sulfur clustered enzymes, inactivates proteins with Fe^2+^ as a cofactor, disrupting iron metabolism, and generating ^•^OH from unbound iron (13). There are many sources of H_2_O_2,_ such as various oxidases including acyl-CoA oxidases and respiratory burst oxidase homologs located in various subcellular compartments (3), but in plants, it is produced primarily during electron transport activity in the chloroplast and superoxide anion dismutation (14).

Where H_2_O_2_ can be beneficial to the plant, O_2_^•−^ is more of a detriment. O_2_^•−^ has a half-life of less than 1 ms and is unable to diffuse far from its site of generation (15). O_2_^•−^ is formed from a one-electron reduction of O_2_, though the radical is not considered a strong oxidizer on its own (Mehler reaction) (Fig. 1*C*). Subsequently, O_2_^•−^ can be converted to H_2_O_2_ by superoxide dismutase (SOD) (Fig. 1*C*). Alternatively, the O_2_^•−^ can interact with Fe^2+^ (found under various forms in plants) to generate the hydroxyl radical (^•^OH) and hydroxide (OH^−^) *via* the Fenton reaction (Fig. 1*C*) (15). There are a variety of sources of O_2_^•−^, the main source being the “electron leakage” from the electron transport chains in the mitochondria and chloroplast (3).

The most unstable and highly toxic ROS, ^•^OH is the most implicated driver of damage caused by ROS. ^•^OH initiates the process of lipid peroxidation and eventual breakdown of organellar and plasma membranes (9). Similar to O_2_^•−^, ^•^OH has a short lifetime and is unable to diffuse far from its site of generation (16). ^•^OH catalyzes the breakdown of polysaccharides and reacts easily with organic molecules (*e.g.*, fatty acids, aromatic compounds, and nucleic acids) to form radical versions of the parent molecule that subsequently react with other neighboring molecules, resulting in cascading oxidative damage (3). As mentioned previously, the Fenton reaction (Fig. 1*C*) forms ^•^OH, but this radical can also be produced through the Haber–Weiss reaction (Fig. 1*C*), where H_2_O_2_ and O_2_^•−^ catalyzed by Fe^2+^ generate OH^−^ and ^•^OH in the mitochondria and cytosol. The Haber–Weiss reaction along with ascorbic acid as a pro-oxidant and high concentrations of glutathione form the ascorbate–glutathione cycle, facilitating ^•^OH production. Both photosystems (PSs) in plants under stress generate abundant amounts of ^•^OH (17).

Along with these ROS, singlet oxygen (^1^O_2_) can act as a powerful oxidizer that reacts rapidly with macromolecules and is a main driver of oxidative damage (5, 9). ^1^O_2_ is the reactive form of O_2_ with antiparallel valence electron spins as compared with ^3^O_2_ (3). Though ^1^O_2_ does not carry a high-energy electron, it can cause rapid oxidative damage to pigments, proteins, lipids, and DNA (6, 8, 18). This is due to its ability to oxidize C–C double bonds and form hydroperoxides or endoperoxides (18) that initiate chain reaction characteristic of ROS-induced oxidative damage. Inhibition of the biosynthesis of the D1 subunit of photosystem II (PSII) correlates with an increase in ^1^O_2_ generation, and this inhibition will then lead to photoinhibition (6). ^1^O_2_ does not diffuse far from its origin on account of its short lifetime, though sufficient stability and ability to diffuse outside the chloroplast and to the cell wall have been reported (19, 20). Because of this lack of diffusion, most ^1^O_2_ reacts in the mesophyll cells of leaves where it is generated from a variety of sources. These sources include the reaction of ^3^O_2_ with light-activated chlorophyll molecules (chlorophyll in its triplet state or ^3^Chl), chlorophyll precursors and catabolites, the cytochrome b6f complex, heme protein and lipoxygenase enzymatic reactions, phytoalexins, hydroperoxides from linoleic acid, and cell wall peroxidase reactions in response to stress (3).

Production of ROS can be caused by many biotic and abiotic stressors, including high light exposure, salt stress, temperature stress, and the application of herbicides (21). During high light stress, chloroplasts and peroxisomes are the major sources of ROS production as the overexcited photosynthetic pipeline produces harmful amounts of ROS (7). Photosynthesis is affected by ROS produced in plastids or produced in other parts of the cell or through phytohormone-related mechanisms (22). Many herbicides can cause ROS accumulation and can act both as primary drivers of damage and as consequences of their primary modes of action (MoA) (23). Here, we will discuss how oxidative damage presents itself under stress caused by herbicidal activity.

Detection of oxidative damage and ROS production can be expressed and measured in many ways. The simplest and most used methods involve quantification of ROS as a whole or individually through colorimetric or fluorescent assays and high-performance liquid chromatography, mass spectrometry, electroparamagnetic resonance spectroscopy, or other analytical procedures (24, 25). Accumulation of malondialdehyde (MDA) is a common marker assessing lipid peroxidation. Another important factor when looking at oxidative stress is the expression and activity of ROS-generating and ROS-quenching enzyme systems. Knowing how this correlates with the accumulation of ROS gives a better picture for the oxidative state of the cell rather than just seeing that ROS are present.

To look at ROS localization in the plant, staining techniques can be employed to visualize where ROS accumulate in different tissues and how that correlates with damage from different stressors. Along with localization, cellular compartmentalization can be observed using fluorescence microscopy and fluorogenic probes specific to different ROS (24). The interpretation of oxidative stress must be multifaceted, looking at multiple forms of evidence to come to a meaningful conclusion. Changes in enzyme activity will be meaningless if ROS levels do not change, thus decreased activity with increased ROS means oxidative stress is present.

Herbicides have been extensively used to understand many cellular and molecular processes in plant biology (26). Herbicidal compounds have high specificity for their target sites and usually inhibit critical biochemical and physiological pathways in the plant. Because of this specificity, herbicides have been very useful in evaluating the downstream repercussions of inhibiting these pathways and shedding insight on how they function (26). This practice of using herbicides to better understand these processes will help us to understand more about the specifics of how ROS play a role in cellular stress.

This review summarizes the involvement of ROS on the MoA of herbicides. It will be divided into the three main categories identified in the new Herbicide Resistance Action Committee (HRAC) classification (https://hracglobal.com/tools/hrac-mode-of-action-classification-2020-map); light activation of ROS, cellular metabolism, and cell division and development (Fig. 2). This new classification map has been approved by the Weed Science Society of America and is now the standard code categorizing herbicide MoAs during registration with the Environmental Protection Agency and on herbicide labels.

**Figure 2:**
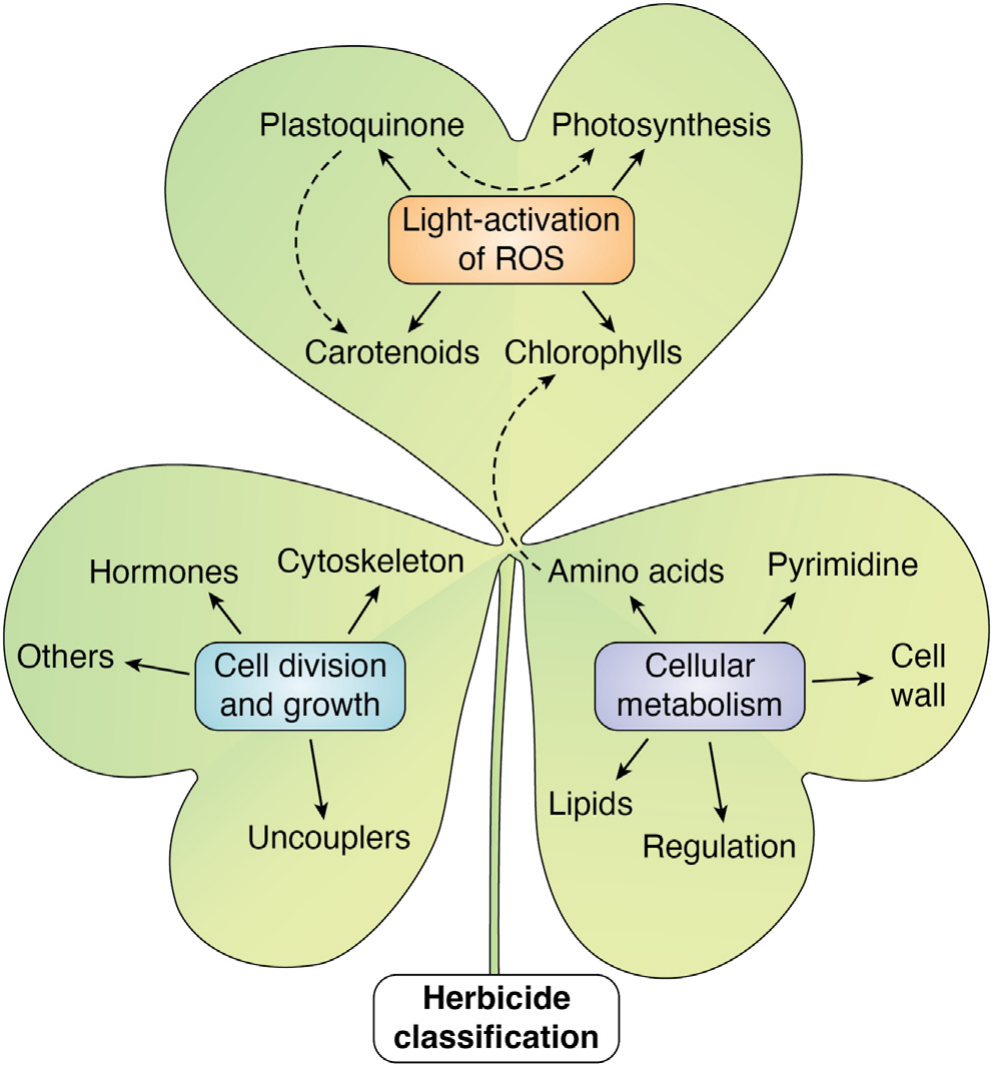
**Summary of the new herbicide classification map from HRAC.** The three main categories are light activation of ROS, cellular metabolism, and cell division and development. Within the light activation of ROS, herbicides target photosynthesis directly, or chlorophylls, carotenoids, or plastoquinone synthesis. Within cellular metabolism, herbicides target amino acids, fatty acids, lipids, pyrimidine, or cell wall synthesis as well as protein regulation. Within cell division and growth, herbicides target cytoskeleton synthesis, hormonal regulation, or other MoA. *Solid arrows* represent direct interactions between herbicides and these processes, whereas *dashed arrows* illustrate indirect effects. HRAC, Herbicide Resistance Action Committee; MoA, mode of action; ROS, reactive oxygen species.

## Light activation of ROS

**Figure 3:**
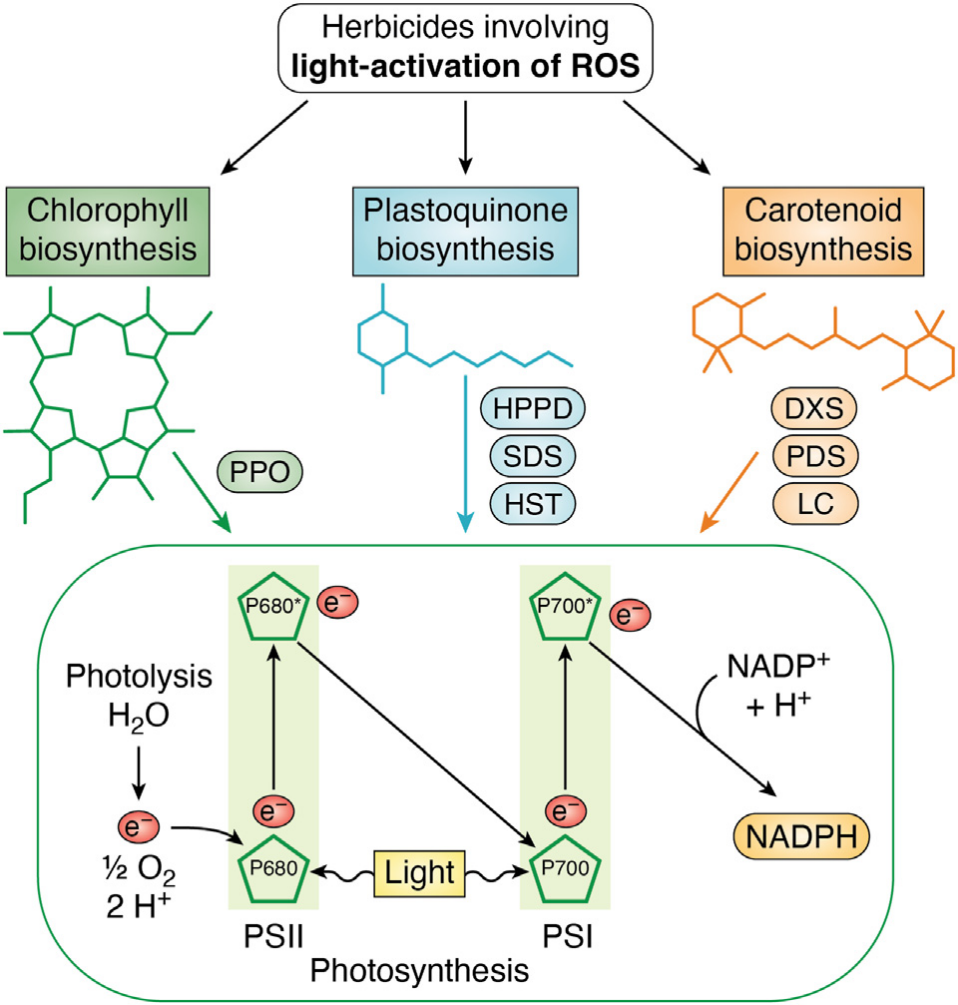
**Summary of HRAC herbicide groups involving light activation of ROS.** DXS, deoxy-d-xylulose phosphate synthase; HPPD, hydroxyphenylpyruvate dioxygenase; HRAC, Herbicide Resistance Action Committee; HST, homogentisate solanesyl transferase; LC, lycopene synthase; PDS, phytoene desaturase; PPO, protoporphyrinogen oxidase; PSI, photosystem I; PSII, photosystem II; ROS, reactive oxygen species; SDS, solanesyl diphosphate synthase.

This section covers the MoAs grouped under light activation of ROS by the HRAC classification. As a group, these herbicides disrupt photosynthesis either by directly inhibiting the photosynthetic electron pathway or targeting the biosynthesis of key electron transport components and pigments (Fig. 3).

### Inhibition of photosynthesis

#### Groups 5 and 6—inhibition of photosynthesis at PSII—serine 264 and histidine 215 binders

PSII herbicides were some of the first synthetic herbicides commercialized. They are divided in two main groups: the serine 264 binders (group 5) and the histidine 215 binders (group 6). Within group 5, there are 27 triazines, 1 triazolinone, 5 triazinone, 5 uracils, 4 phenylcarbamates, 2 pyridazinones, 27 ureas, and 2 amides, whereas group 6 includes 3 nitriles, 1 phenylpyridazine, and 1 benzothiadiazinone (https://hracglobal.com/tools/hrac-mode-of-action-classification-2020-map). The diversity of herbicides targeting PSII suggests that their target is very promiscuous. These herbicides are selective (control weeds without injuring crops) and can be applied either to soil and/or to foliage and used at relatively high rates because of the very high number of binding sites in photosynthetic tissues (27). The initial urea- and triazine-type herbicides are still the most common classical PSII inhibitors. However, another class of phenolic-type PSII inhibitors was introduced in 1979 (*e.g.*, ioxynil and bromoxynil). These compounds are potent blockers of photosynthetic electron flow. While the classical and the phenolic-type herbicides interacts with PSII differently, they both interfere with binding of the mobile electron (and proton) carrier plastoquinone (PQ) to the Q_B_ site of the D1 protein (28). Preventing PQ reduction at Q_B_ has many catastrophic consequences, including rapid interruption of CO_2_ fixation, reduction of D1 protein turnover, toxic nitrite accumulation in the chloroplast, loss of photoprotective antioxidative compounds (*e.g.*, ascorbate and carotenoids), and photodegradation of chlorophylls. Under full sunlight, treated leaves wilt and die within a few days (27).

PSII is a multisubunit enzymatic complex embedded in the thylakoid membranes and a key component of the photosynthetic apparatus. The first step in photosynthesis begins at the level of PSII and involves splitting of H_2_O to produce O_2_ by PSII. This process generates reducing power in the form of electrons to produce ATP and NADPH as part of the light reaction of photosynthesis. The electrons are channeled by a photosynthetic electron transport chain composed of several proteins that are associated with small redox type molecules such as PQ. The D1 protein of PSII is part of this electron transport complex. The Q_B_-binding site of D1 normally binds PQ, which is reduced by two electrons originating from the water-splitting reaction of PSII. Plastohydroquinone is reoxidized by the cytochrome b_6_f complex involving a proton-motive Q cycle (27, 29). Structure–activity relationship studies revealed that herbicides with the highest affinity for the D1 Q_B_ site form hydrogen bonds with His215, whereas weaker inhibitors lack specificity for the Q_B_ (30). These interactions were confirmed with analyses of crystal structure of PSII with a bound herbicide (31).

Under normal circumstances, a one-electron redox shift of PQ/semiquinone (Q_A_/Q_A_^•^) allows for safe PSII charge recombination reactions and protects PSII from photodegradation by minimizing the amount of ^1^O_2_ generated by ^3^Chl (32). An important aspect of the MoA of PSII inhibitors is that these herbicides prevent the redox equilibration between the unstable (reactive) plastosemiquinone and PQ and the PQ bound at site B, resulting in toxic accumulation of ^1^O_2_ (33).

In many biological systems, free H_2_O_2_ can be reduced by metals (Fe^2+^ > Mn^2+^ > Cu^+^, listed in order of reducing strength) to form ^•^OH *via* the Fenton reaction (Fig. 1*C*). Spin trapping electroparamagnetic resonance spectroscopy technique demonstrated that the production of ^•^OH by thylakoid membranes is enhanced in the presence of these metals, suggesting that ^•^OH is formed by one-electron reduction of H_2_O_2_ *via* the Fenton reaction (Fig. 1*C*). Similar experiments demonstrated that PSII inhibitors produced large amounts of ^•^OH. Furthermore, the production of ^•^OH caused by diuron, a urea-type PSII-inhibiting herbicide, was reduced in the presence of exogenous catalase (CAT), confirming the involvement of H_2_O_2_ (34). Phenolic herbicides induce stronger formation of ^3^Chl and ^1^O_2_ than urea herbicides (35). The stimulated charge recombination induced in herbicide-treated PSII is an important contributor to the herbicidal action mediated by ^1^O_2_ (36). This is supported by the following observations.

Phenolic herbicides cause greater photodamage than the urea- and triazine-type herbicides although all these herbicides bind to the Q_B_-binding site and inhibit forward electron transport. Different classes of herbicides affect photosynthetic charge recombination differently, with phenolic herbicides lowering the emission temperature, whereas classical herbicides increase it. Indeed, compounds binding to the D1 protein alter the midpoint potential (*E*_m_) of the Q_A_/Q_A_^•^ redox couple, with phenolic herbicides lowering the *E*_m_ by 45 mV, whereas diuron raises the potential by 50 mV. While ^1^O_2_ is clearly generated by PSII inhibitors, photoinhibitory damage of PSII produces O_2_^•−^ and ^•^OH (35).

Light-induced charge pairs induced by PSII inhibitors decay by a charge recombination route involving the formation of the primary P680/pheophytin radical pair (P680^+•^Pheo^−•^) and of a ^3^Chl reacting with O_2_ to form ^1^O_2_ (36). There are two competing recombination pathways in PSII caused by herbicides inhibiting photosynthetic electron transport. Since the midpoint potential of Q_A_ is lowered by about 50 mV by phenolic herbicides, the charge recombination pathway *via* the primary radical pair is favored. On the other hand, classical PSII inhibitors (*e.g.*, atrazine and diuron) increase the midpoint potential of Q_A_ by about 100 mV resulting in lower amount of ^1^O_2_ formation because a safer recombination route is favored minimizing the formation of the primary radical pair and the formation of ^3^Chl and ^1^O_2_. In addition, evidence supporting the involvement of two competing pathways was observed in PSII with mutations either on the electron donor side (37) or on the electron acceptor side (38) that affect the energy gap between P680^+•^Q_A_^•−^ and P680^+•^Pheo^−•^.

#### Group 22—electron diversion at photosystem I

The first group 22 herbicides were discovered in the 1950s (*e.g.*, paraquat [PQT] and diquat), but other pyridinium actives include cyperquat and morfamquat (27). These compounds are nonselective and have been used extensively for control and management of terrestrial and aquatic vegetation, including crop desiccation, pasture renovation, crop production with limited or no tillage, and total weed control (39).

Group 22 herbicides are classified as photosystem I (PSI) electron diverters. While PSII inhibitors induce chlorosis and necrosis over several days, group 22 herbicides develop symptoms within a few hours after treatment. Initial symptoms are the occurrence of dark green areas on treated leaves. These nonselective herbicides are the most rapidly acting contact herbicides, causing severe wilting and desiccation of the foliage within an hour after treatment. Since the effects are so rapid, these compounds normally have limited abilities to translocate to other tissues.

The PSI complex consists of the components of photosynthetic electron transport that utilizes the electrons generated by the water-splitting complex of PSII (40) to catalyze the reduction of ferredoxin with plastocyanin as the electron donor (41). This provides energy to reduce NADP^+^ to NADPH.

The MoA of all group 22 herbicides is identical; therefore, PQT will be used as a model molecule. First, bipyridinium herbicides assume coplanar configurations with highly conjugated π-electron systems. These properties can stabilize the unpaired electron of the reduced free radical form of the molecule, which is essential for the reduction of O_2_ *via* the following mechanism: PQT (methyl viologen) is applied in its colorless divalent cation PQT^2+^ form. Because PQT and other bipyridinium herbicides have redox potential between −300 and −700 mV, they can divert electrons from the iron–sulfur center PSI. PQT^2+^ is reduced to its intensely blue monovalent cation radical PQT^+•^ by PSI. PQT^+•^ is highly reactive and reduces O_2_ to O_2_^•−^, and PQT^2+^ is regenerated. SOD provides some protection against PQT by converting O_2_^•−^ to H_2_O_2_ and O_2_. PQT^+•^ condenses with H_2_O_2_ to spontaneously produce PQT^2+^, ^•^OH, and OH^−^. ^•^OH can be produced by the Fe^+^-dependent Fenton reaction (Fig. 1*C*) (42). Consequently, electron diverters produce massive amounts of O_2_^•−^ and other reactive radicals, leading to massive lipid peroxidation and loss of membrane integrity (43). When isolated chloroplasts are treated with a bipyridinium herbicide, the auto-oxidation by O_2_ is observed as a light-dependent oxygen consumption or Mehler reaction (Fig. 1*C*) (44, 45).

H_2_O_2_ is often produced in nontreated plants and plays important roles in plant biology (12). However, it is readily dissipated *via* the ascorbate–glutathione cycle (46). However, the amount of O_2_^•−^ and H_2_O_2_ generated following exposure to PQT overwhelms the native antioxidative protection mechanisms. Consequently, these ROS ultimately react with each other to produce ^•^OH *via* the Haber–Weiss reaction (Fig. 1*C*) (47, 48). ^•^OH are the most potent biological oxidants known and quickly and effectively initiate membrane degradation through lipid peroxidation of polyunsaturated fatty acids (43).

Antagonism between PSII and PSI inhibitors is common because PSI depends on both the electrons and oxygen generated by PSII. Therefore, PSII inhibitors reduce the electron flux going toward PSI and the amount of oxygen released by the water splitting complex, preventing the reduction of PSI inhibitors and the subsequent generation of ROS.

### Inhibition of pigment biosynthesis

#### Group 14—inhibition of protoporphyrinogen oxidase

Protoporphyrinogen IX oxidase (PPO or protox)–inhibiting herbicides are effective tools to control a broad spectrum of weeds. PPO inhibitors cause rapid photobleaching and light-dependent desiccation of foliage (49). PPO (Enzyme Commission number: 1.3.3.4) catalyzes the oxidation of protoporphyrinogen (protogen) to protoporphyrin IX (proto), the last common precursor of the tetrapyrrole pathway. The subsequent reactions are catalyzed by either an iron or a magnesium chelatase that lead to the formation of Fe-proto or Mg-proto, the key precursors of heme and chlorophyll, respectively (50, 51). PPO is the molecular target site for several chemical classes (*e.g.*, diphenyl ethers, phenylpyrazoles, *N*-phenyloxadiazolones, *N*-phenyltriazolinones, and *N*-phenylimides) (52). Strangely, inhibition of PPO causes a rapid and unregulated extraplastidic accumulation of protos responsible for the contact activity of these molecules (53, 54, 55, 56, 57, 58). This MoA confounded scientists for years. However, it is now understood that inhibition of PPO deregulates porphyrin biosynthesis and causes protogen (colorless substrate) to accumulate and leak into the cytoplasm where it is rapidly oxidized nonenzymatically to the highly photodynamic proto. Photoexcitation of proto present in the cytosol causes a burst of ROS that destabilize membranes *via* lipid peroxidation (23, 59, 60, 61). The catastrophic consequences of the unregulated accumulation of protoporphyrin in the cytosol and subsequent generation of ROS have secondary effects on the stability of PSI and PSII reaction centers and other processes (62).

There is a direct relationship between the amount of proto accumulating and the loss of membrane integrity (53). While the selectivity of these herbicides is mostly because of differential abilities to metabolize the active ingredients, some plants have additional natural tolerance to PPO inhibitors by having strong chemical and enzymatic antioxidant systems that protects them against the ROS generated by PPO inhibitor action. Hydrophilic antioxidants (*e.g.*, reduced glutathione and ascorbic acid) were particularly strong protectants (63).

### True bleaching herbicides

**Figure 4:**
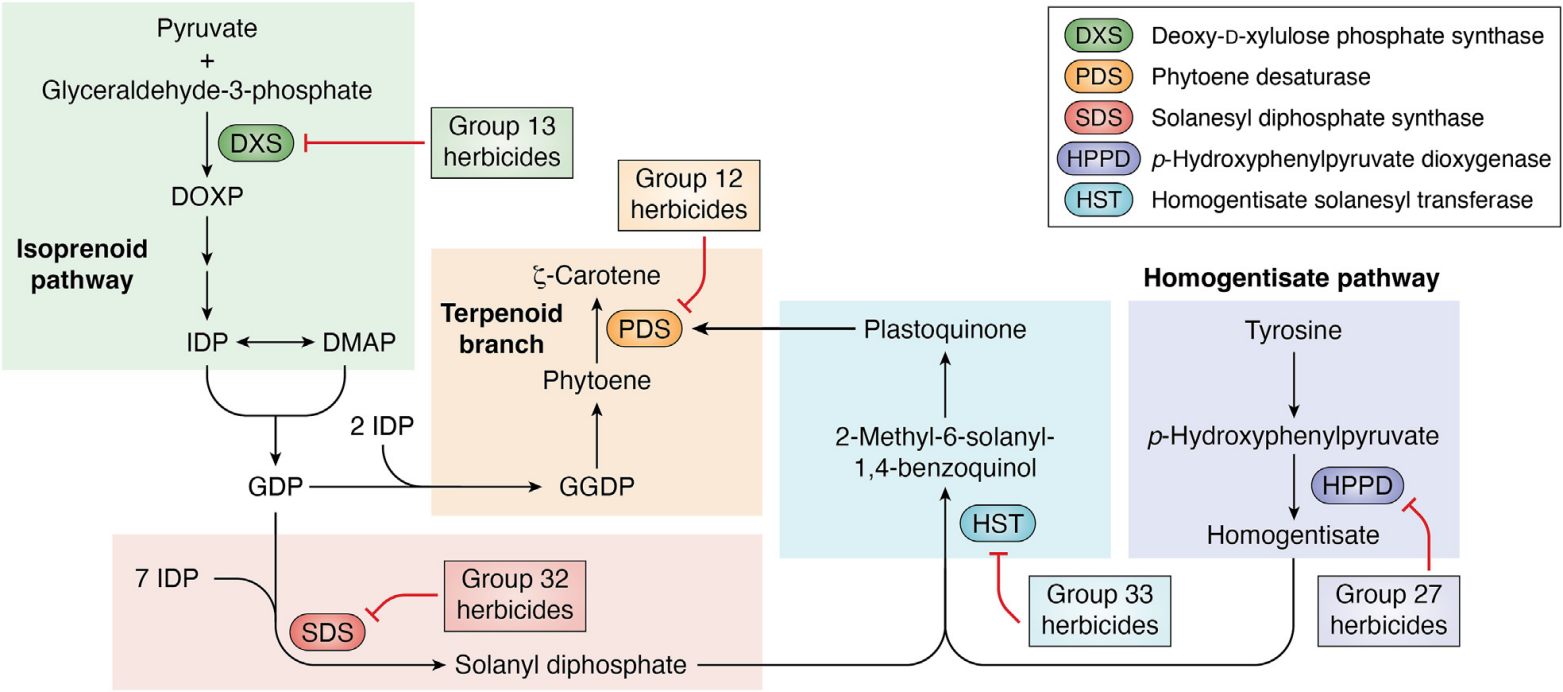
**Overview of herbicides inhibiting carotenoids and plastoquinone biosynthesis.** The isoprenoid pathway (*green box*) begins with the formation of deoxy-d-xylulose phosphate (DOXP) by the action of DXS. This pathway ultimately leads to the formation of isopentenyl diphosphate (IDP). The condensation of IDP with dimethyl allyl diphosphate (DMAP) initiates the terpenoid branch (*yellow box*) that can be directed toward carotenoid biosynthesis, with PDS catalyzing a key step. Terpenoid biosynthesis can lead to the formation of solanesyl diphosphate (*red box*), the lipophilic tail of plastoquinone *via* the action of SDS. The benzoquinone head of plastoquinone is derived from the homogentisate pathway (*purple box*) involving the key enzyme HPPD. The complete plastoquinone structure is assembled by transferring the solanesyl tail to the homogentisate head (*blue box*) by the action of HST. The targets of herbicide groups affecting carotenoid and/or plastoquinone biosynthesis are included. DXS, deoxy-d-xylulose phosphate synthase; HPPD, hydroxyphenylpyruvate dioxygenase; HST, homogentisate solanesyltransferase; PDS, phytoene desaturase.

Several classes of herbicides bleach leaf tissues (loss of carotenoids and chlorophylls) as their primary symptom of their MoA. Therefore, these will be discussed as a separate group, based on where the target site is located in the overall biosynthesis scheme (Fig. 4). Because of the complexity of the relationship between these bleaching herbicides, inhibitors directly involved in plastidic terpenoid and carotenoid biosynthesis will be covered first. Compounds causing bleaching indirectly by targeting PQ biosynthesis will be discussed next.

A perplexing aspect of the MoA of herbicides targeting carotenoid biosynthesis is their general lack of ability to induce ROS accumulation. Since one of the main functions of carotenoids is to quench ROS produced by photosynthesis when plants are exposed to excess light, one would expect that inhibition of carotenoid biosynthesis would cause catastrophic ROS accumulation from the “unprotected” PSs; however, that is not the case. A possible explanation is that the proteins involved in PSII, most notably the D1 protein, have extremely rapid turnover, and the two reaction-center β-carotenes are required for the reassembly of the PS. Consequently, PSII stops functioning before total pigment bleaching, thus preventing the uncontrolled generation of ROS by photosynthesis in the absence of carotenoids (64). Therefore, regardless of the target, perhaps with the exception of group 32 inhibited by aclonifen, the MoA of most compounds affecting carotenoid biosynthesis does not directly involve light-dependent ROS-driven tissue injury (23). Consequently, these herbicides should not be classified by HRAC under the category light activation of ROS.

### Bleaching herbicides directly targeting plastidic terpenoid biosynthesis

#### Group 12—inhibition of phytoene desaturase

Phytoene desaturase (PDS) catalyzes a key early step in carotenoid biosynthesis in plants. Carotenoids are important pigments involved in photosynthesis and act as antioxidants that protect plants from ROS produced during photosynthesis. However, as with group 13 herbicides, group 12 herbicides that target PDS (*e.g.*, picolinafen, flurtamone, flurochloridone, diflufenican, norflurazon, and fluridone) strongly inhibit carotenoid biosynthesis but have no direct peroxidative activities (65). That is because loss of carotenoids leads to the destruction of antenna chlorophyll and irreversible PSII inactivation (as described in the introductory paragraph of the “True bleaching herbicides” section), resulting in rapid bleaching of young leaves but not rapid necrosis (23, 66, 67, 68, 69). Additional factors protecting from the potentially harmful ROS species generated by group 12 herbicides include increases in antioxidant enzyme activity (*e.g.*, peroxidase, SOD, glutathione reductase [GR], and ascorbate peroxidase [APOX]) (70).

#### Group 13—inhibition of deoxy-d-xylulose phosphate synthase

Group 13 herbicides (*e.g.*, clomazone and bixlozone) inhibit deoxy-d-xylulose phosphate synthase. This enzyme catalyzes the first step in the isoprenoid pathway, and its inhibition prevents the formation of all plastidic terpenoids. Without carotenoids, the treated tissues rapidly lose their chlorophylls and their ability to generate O_2_ from water and photosynthetic electron flow, which accounts for the relatively low ROS damage and subsequent membrane degradation caused by these herbicides (23). While clomazone can induce some ROS production and result in oxidative stress and moderate cell damage in plants that have low capacity to quench these ROS (71), ROS production can be greatly increased by mixing group 13 inhibitors with inhibitors of PSII (groups 5 or 6) (67). Synergistic responses with inhibitors of PSII have also been reported with fosmidomycin, a herbicidal compound that targets the enzyme DOXP reductoisomerase, an enzyme catalyzing the reaction step after deoxy-d-xylulose phosphate synthase.

### Bleaching herbicides targeting PQ biosynthesis

#### Group 27—inhibition of hydroxyphenylpyruvate dioxygenase

As opposed to group 32 (discussed later), hydroxyphenylpyruvate dioxygenase (HPPD) inhibitors have no direct effect on plastidic terpenoid biosynthesis. Instead, they inhibit PQ biosynthesis. This has multiple consequences on plant physiology, as PQ is required for both photosynthetic electron transport and carotenoid biosynthesis, being an essential redox cofactor of PDS activity (72). However, the injury symptoms are more strongly associated with the bleaching of leaf tissue observed with other bleaching herbicides, rather than inhibition of photosynthesis and its associated rapid ROS-dependent loss of membrane integrity. However, HPPD inhibitors can be synergized by group 5 inhibitors (PSII), resulting in rapid tissue necrosis, and increased herbicidal response in comparison to either herbicide applied alone (73, 74, 75). The increased herbicidal activity from these mixtures is due to the biochemical relationship between the two targets. These herbicide mixtures involve a concurrent reduction in PQ and α-tocopherol associated with HPPD inhibition, allowing for more effective inhibition of photosynthetic electron transport because the PSII-inhibiting herbicide does not have to compete with PQ at their mutual binding site on the D1 protein (67, 74).

#### Group 32—inhibition of solanesyl diphosphate synthase

Group 32 herbicides are at the interphase between those that inhibit plastidic terpenoid biosynthesis directly and those that target PQ biosynthesis, resulting in indirect inhibition of carotenoids. Solanesyl diphosphate synthase catalyzes the formation of solanesyl diphosphate, a very long terpenoid that later forms the lipid tail of PQ. Inhibition of this enzyme by the diphenyl ether herbicide aclonifen causes bleaching of treated plants because PQ is an essential redox cofactor for PDS activity (72, 76). While inhibition of PQ biosynthesis generally does not lead to catastrophic ROS formation, aclonifen has been reported to weakly inhibit PPO (group 14). Consequently, it can induce ROS formation in the same manner described for group 14 herbicides but not as effectively as herbicides that act primarily on PPO. For example, aclonifen caused ROS accumulation of treated leaves but not as much more potent PPO inhibitors (77). As previously mentioned, group 32 herbicides specifically inhibit the formation of solanesyl diphosphate, a terpenoid. However, solanesyl diphosphate is the precursor of the lipophilic polyprenyl tail of PQ, and herbicides targeting solanesyl diphosphate synthase are lethal because of their inhibition of PQ biosynthesis.

#### Group 33—inhibition of homogentisate solanesyltransferase

As mentioned previously, PQ biosynthesis involves the convergence of homogentisate and terpenoid biosynthesis pathways. Homogentisate solanesyltransferase (HST) catalyzes this step by attaching the long terpenoid solanesyl to homogentisate to form the first intermediate in PQ biosynthesis (78). Herbicides targeting HST (*e.g.*, cyclopyrimorate) cause similar bleaching of young plant tissues. Cyclopyrimorate is a proherbicide, so its desmorpholinocarbonyl cyclopyrimorate metabolite is the active form inhibiting HST (79). As with other bleaching herbicides, the herbicidal activity of HST inhibitors does not rely primarily on ROS accumulation (80). Since HST is downstream from the reaction catalyzed by HPPD in the PQ biosynthesis, the herbicidal activity of cyclopyrimorate is enhanced in tank mix with group 27 herbicides.

#### Group 34—inhibition of lycopene cyclase

Early work on amitrole recognized that this herbicide causes bleaching of photosynthetic tissues. It was first suggested that this herbicide may become a free radical (similar to group 22 herbicides) (81) and then proposed that it inhibited histidine biosynthesis (82). It was later demonstrated that amitrole bleaches newly developing shoot tissues, but its herbicidal activity does not involve ROS formation, inducing only small amount of necrosis. Amitrole hinders chloroplast development with little visible effect on other plant parts (83). Though the MoA of amitrole has been difficult to elucidate, its symptomology is similar to other bleaching herbicides, with loss of carotenoids and the consequent photodestruction of chlorophyll and chloroplast disruption (84), including reduction of the thylakoid system and destruction of most 70S ribosomes (85, 86). HRAC now classifies amitrole as an inhibitor of lycopene cyclase (LC). However, there is still some uncertainty on the actual target site of this herbicide. Indeed, it is now clear that amitrole does not block histidine biosynthesis or pigment biosynthesis (87). Consequently, it should not be considered to act primarily as an inhibitor of LC (66, 88). Furthermore, compounds that target LC are generally weakly herbicidal (66).

### Unexpected light activation of ROS involving amino acid biosynthesis

#### Group 10—inhibition of glutamine synthetase

Glufosinate-ammonium (2-amino-4-(hydroxymethylphosphinyl)butanoic acid) is a d/l racemic mixture of the natural product l-phosphinothricin produced by certain *Streptomyces* spp. (89). Glufosinate is the only commercial herbicide targeting glutamine synthetase (GS) (27). GS is the second most abundant protein in plant leaves that plays a central role in nitrogen metabolism by incorporating ammonia into glutamate to yield glutamine (90, 91). While glutamine synthase plays a key role in amino acid biosynthesis, and as such should belong to the “Cellular metabolism” section, inhibition of GS induces a catastrophic accumulation of ROS in the presence of light. Therefore, it is included in this section.

As a potent competitive irreversible inhibitor of GS, glufosinate disrupts photorespiration, resulting in a rapid reduction of glutamine levels and ammonia accumulation (92). However, the exact sequence of events leading to the rapid desiccation of the foliage associated with this herbicide has remained elusive for many years. If depletion of the glutamine pool levels was the phytotoxic factor driving the MoA of glufosinate, plants would exhibit a slow death similar to what is commonly associated with herbicides targeting branched chained and aromatic amino acid biosynthesis. The dramatic rise in ammonia levels resulting from the disruption of photorespiration (93) has been proposed as the main cause for the fast response to glufosinate (94, 95). Furthermore, glufosinate affects carbon assimilation (96, 97) by altering the photorespiratory processes.

While ammonia accumulation is a physiological consequence of GS inhibition, we recently demonstrated that it is not the cause of the rapid necrosis of the foliage. Indeed, glufosinate-treated plants exogenously supplied with glutamine-accumulated high levels of ammonia, but no injury was visible if the plants were maintained under low light (98, 99). Furthermore, ammonia accumulated in both mature and young leaves, but rapid phytotoxicity was observed only in older leaves, suggesting that tissues must be fully photosynthetically active for the injury to occur. Finally, reducing photosynthetic electron transport with low doses of atrazine protected the plants against developing glufosinate-dependent desiccation of the leaf tissues while still accumulating high levels of ammonia (99).

It is now established that a massive light-dependent production of ROS causing catastrophic lipid peroxidation of the cell membranes is responsible for the rapid desiccation observed in plants treated with glufosinate (98, 99). A recent study describes the MoA of glufosinate as follows. Glufosinate first inhibits GS, disrupting photorespiration and causing ammonia, glycolate, and glyoxylate accumulation (95, 99), which in turn inactivates the Calvin cycle (100). The combined disruption of photorespiration and photosynthetic carbon assimilation leads to a catastrophic light-dependent burst of oxidative stress in the chloroplasts.

## Cellular metabolism

**Figure 5:**
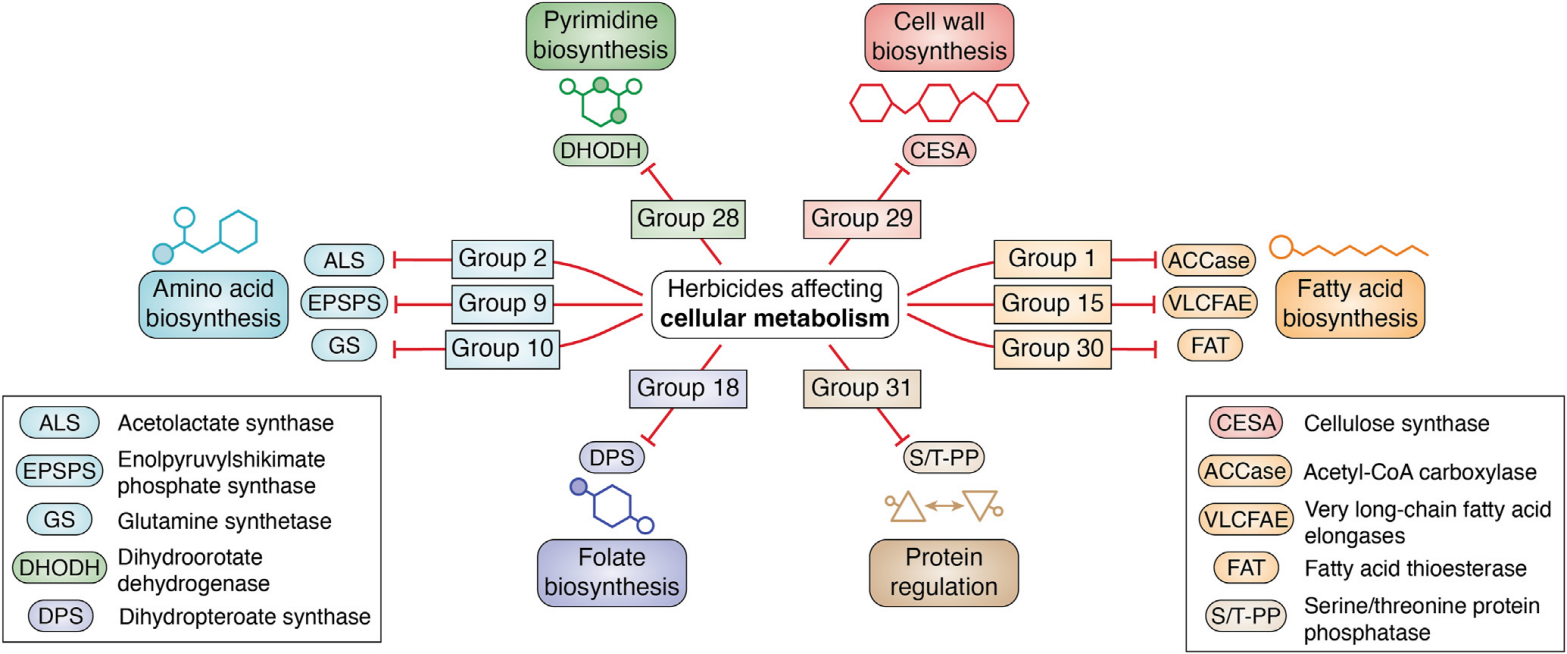
**Summary of HRAC groups affecting cellular metabolism.** These herbicides target various aspects of metabolism, including amino acids, pyrimidine, cell wall, fatty acids, folate biosynthesis, or protein regulation. The targets of herbicide groups affecting these pathways are included. HRAC, Herbicide Resistance Action Committee.

This section covers herbicides classified by HRAC as inhibitors of cellular metabolism. These molecules target either the synthesis of key biochemical building blocks (amino acids, fatty acids, and nucleotides) or their assembly in macromolecules (proteins and cellulose) needed for regular cellular function (Fig. 5).

#### Group 1—inhibition of acetyl coenzyme A carboxylase

Acetyl coenzyme A carboxylase (ACCase) inhibitors block the first step in plant lipid biosynthesis by inhibiting the carboxytransferase partial reaction of ACCase activity. This inhibition is both rapid and concentration dependent. Disruption of fatty acid biosynthesis has many detrimental effects on plants as the downstream products of this pathway include many unique fatty acids that have crucial structural and biochemical roles (101, 102). Disruption of fatty acid biosynthesis can also lead to oxidative damage through lipid peroxidation in response to the production of many ROS species.

Clethodim treatment caused changes in maize antioxidant system response and lipid peroxidation level (103). These plants had elevated levels of H_2_O_2_ and MDA in treated seedlings and a significant increase in peroxidase activity in response to clethodim. The cytotoxic and physiological effects of fluazifop-P-butyl on the chloroplast, photosynthetic pigment, oxidative stress, and activity of antioxidative enzymes were investigated in peanut leaves by Fayez *et al.* (104). This herbicide caused a degeneration of thylakoids and a decrease in photosynthetic pigment contents, which could be due to enhanced production of ROS and oxidative stress. Fluazifop-P-butyl injuries to peanut leaves were associated with enhanced lipid peroxidation and photosynthetic pigment degradation. There was an increase in MDA with increasing fluazifop-P-butyl concentrations but with more variable levels of H_2_O_2_. The activity of CAT, peroxidase, and APOX increased after fluazifop-P-butyl treatment, whereas SOD activity decreased. SOD is a major scavenger of O_2_^•−^ and other ROS, thus this reduction in SOD activity indicates that ROS was not scavenged sufficiently, causing oxidative stress in peanut leaves.

The evolution of ROS-induced phytotoxic action in ACCase inhibition was investigated on *Acanthospermum hispidum* treated with fluazifop-P-butyl herbicide (105). Fluazifop-P-butyl increased MDA content in a concentration-dependent manner in *A*. *hispidum*. Their results suggest that ROS induced by fluazifop-P-butyl has a phytotoxic role in this plant, and that peroxidation of membrane lipids by ROS is part of the primary action of fluazifop-P-butyl in *A*. *hispidum*. Overall, many studies support the observations that ACCase inhibition is accompanied by oxidative damage by showing that the use of ACCase inhibitors increases antioxidant enzyme activity and overproduction of many ROS species.

#### Group 2—inhibition of acetolactate synthase

Acetolactate synthase (ALS) inhibitors represent the second largest class of herbicides and control a wide spectrum of annual and perennial grasses and broad-leaf weeds. These compounds inhibit synthesis of branched amino acids and are slow acting, causing wilting and ultimately plant death (102). ALS inhibition is associated with an oxygen-consuming side reaction that has brought up the question of whether oxidative damage contributes to their MoA (101). The potential involvement of ROS in the mechanism of ALS inhibition was first proposed in 1994 (106). They had detected an oxygenase reaction during this inhibition with a result of ^1^O_2_, which was itself inhibited at high concentrations of ALS. The study showed that there was involvement of ROS in the irreversible damage of ALS by these herbicides and posited that these herbicides should increase oxidative inactivation and that this could be a result of the MoA being directly involved in irreversible oxidative damage of ALS. The claims that ROS were directly involved in the mechanism of ALS inhibition were refuted in 2007 (107). Lipid peroxidation detected after treatment with ALS herbicides did not affect membrane integrity, and there were no important changes to antioxidant enzymes. The potential oxidative stress triggered was not severe enough to cause changes in antioxidant machinery, though this lack of response could be due to treated plants inability to respond. This study claimed that this oxidative stress is a secondary consequence of ALS inhibition.

Many studies have looked at the role of ROS and oxidative damage in ALS inhibitors outside its primary mechanism. Metsulfuron-methyl, an ALS-inhibiting herbicide, when applied to wheat and wild radish, resulted in less accumulation of H_2_O_2_ compared with the control 120 h after treatment and led to MDA accumulation (108). Oxidative stress caused by metsulfuron-methyl was similar or lower than the control samples. Broad bean and maize treated by rimsulfuron accumulated H_2_O_2_ within 4 to 8 days after treatment followed by a sharp decline (109). SOD activity levels also increased transiently under rimsulfuron treatment and then returned to control levels. Consequently, it was concluded that the MoA of rimsulfuron does not involve the generation of oxidative stress in either of these species. In rice plants treated with penoxsulam, there was a significant increase in H_2_O_2_, SOD activity, and thiobarbituric acid reactive substances, a common measure of lipid peroxidation using MDA as reference, at 96 h after treatment (110). These treated plants had a lower CAT activity 48 and 96 h after treatment. These results indicate that penoxsulam has a greater potential to cause oxidative stress in rice. While studying the enantioselectivity of imazethapyr in *Arabidopsis*, a decrease in the transcription levels of CuSODs and an increase in the transcription levels of FeSODs (111) were noted. On the other hand, the activity of antioxidants SOD, CAT, and glutathione peroxidase decreased with increasing MDA levels. It was concluded that imazethapyr suppressed the ROS scavenging system and enhanced ROS production.

One of the largest sources of ROS in plant stress derives from damage to chloroplasts and interference with photosynthesis. In rapeseed, treatment with sulfonylurea and other ALS-inhibiting compounds resulted in the destruction of chloroplasts and photosynthesis incapability (112). Mutant Arabidopsis strains of the proton gradient regulation 5 protein and chloroplast NAD(P)H dehydrogenase were used to explore photosynthetic metabolic pathways after treatment with imazethapyr (113). The effect on photosynthesis metabolic pathways in NAD(P)H dehydrogenase and proton gradient regulation 5 mutant Arabidopsis treated with imazethapyr had a significantly higher level of H_2_O_2_ and O_2_^•−^ 6 days after treatment. This increase in ROS accumulation across all treated groups indicates greater oxidative stress under imazethapyr. The PSII system was substantially inhibited after treatment, thus resulting in a negative impact on photosynthesis metabolic pathway that will result in ROS overproduction. Overall ALS inhibition seems to have some effect on oxidation and ROS accumulation in treated plants but to an extent that is secondary to the damage caused by ALS inhibition. The presence of oxidative damage and ROS accumulation in treated plants can be attributed to a secondary effect of stress in plants.

#### Group 9—inhibition of 5-enolpyruvylshikimat-3-phosphate synthase

Glyphosate is one of the most used and well-known herbicides currently on the market. It acts by inhibiting 5-enolpyruvylshikimate-3-phosphate synthase (EPSPS), an important enzyme in the shikimic acid pathway involved in the production of aromatic amino acids. Glyphosate depletes aromatic amino acid levels and compromises biosynthetic metabolic pathways in plants (102). Unlike the other herbicide group that affects amino acid biosynthesis, ALS inhibitors, there is no known direct connection between glyphosate and EPSPS and any ROS-producing processes. Though the process is unknown, many studies have reported that treatment with glyphosate results in altered oxidative states in plants. Glyphosate can affect physiological processes that often lead to ROS as shown by the reducing effect on mitochondrial electron transport chain in *Dimorphandra wilsonii* embryos treated with glyphosate (114). Willow plants treated with glyphosate had decreased PSII quantum yields and accumulated H_2_O_2_.

Studies across a vast array of organisms have investigated the specific physiological effects of the toxicity of glyphosate. Among these many physiological effects, glyphosate alters oxidative states in treated plants (115, 116, 117, 118, 119, 120, 121, 122, 123, 124, 125, 126, 127, 128, 129, 130). This oxidative stress is often associated with an accumulation of H_2_O_2_ and other ROS and commensurate increases in expression or activity of many antioxidative enzymes (115, 116, 117, 118, 119, 121, 122, 125, 126, 127, 128, 129, 131, 132). Though this physiological response is common, some studies reported little to no difference in ROS accumulation or changes in antioxidant activity after glyphosate treatment (114, 118, 124, 128, 130, 133, 134) or even decrease in ROS levels and antioxidant activity, resulting in an altered oxidative state but in the opposite direction (118, 120, 121, 129, 130). Overall, there is much evidence that glyphosate can result in an altered oxidative state in treated plants as a secondary effect.

Many studies have investigated how ROS affects glyphosate resistance and how some glyphosate-resistant populations have enhanced antioxidant potentials along with target site resistance, conferring higher resistance to glyphosate (132, 133, 134, 135, 136). Glyphosate-resistant *Amaranthus palmeri via* EPSPS gene amplification were used to test multiple oxidative stress damage markers, ROS content, and antioxidant activity in leaves of two *A. palmeri* populations treated with different glyphosate doses (135). A dramatic 250% increase in ROS levels was observed in the susceptible (S) population, relative to untreated plants, and accumulation was unaffected by glyphosate dose. No changes occurred in the resistant (R) population comparing untreated and treated. The study determined that lack of protein carbonylation, a typical oxidative damage marker, and the dose-dependent lipid peroxidation suggested that moderate oxidative damage was not the cause of death after glyphosate application (135). The R population did neither die nor accumulate ROS or incur oxidative stress, thus this glyphosate resistance trait was thought not to coincide with increased tolerance to oxidative stress and that glyphosate-induced oxidative stress comes from EPSPS inhibition. It was postulated that peroxidase activity could be a source of ROS because of it being the sole source of increased enzymatic activity in the S population in response to glyphosate (135). Overall, the study concluded that EPSPS inhibition caused mild oxidative stress in *A. palmeri* as a secondary effect caused by glyphosate. A metabolomic study done on glyphosate-resistant *A*. *palmeri* that measured the final products of gene expression, protein expression, and enzymatic activity confirmed that ROS were a secondary effect of glyphosate treatment and not the main driver of plant damage caused by glyphosate treatment (136). The resistant biotype of *A. palmeri* with lower EPSPS copies had an enhanced antioxidant potential because of the production of ROS in tissues, when compared with individuals with greater EPSPS copy numbers.

#### Group 29—inhibition of cellulose synthesis

Inhibition of cellulose biosynthesis can lead to weakened cell walls, abnormal or restricted growth, and subsequent plant death (101). This is because cellulose is an integral part of the plant cell wall and is necessary for cell wall synthesis (101). The cell wall is naturally involved in stress response, in particular with ROS signaling, as said signaling is necessary for cell division by way of cell wall loosening (137). This involvement with ROS and oxidation in the natural function of the cell wall leads to the natural conclusion that treatment with cellulose biosynthesis inhibitors (CBIs) should result in a modified oxidative system in plant cells. CBIs do not have a common site of action in the synthesis of cellulose (101), and though this group may not have a common site of action, there are commonalities in consequences of inhibition of cellulose biosynthesis, and production of ROS is often observed with these herbicides.

The CBI isoxaben has been used in many studies to investigate cell wall synthesis and dynamics. In these studies, treatment with isoxaben upregulated stress response genes, production of ROS, accumulation of phytohormones, changes in cell wall composition, and growth arrest (138, 139, 140, 141, 142). Cell wall maintenance and damage involves signaling *via* many receptor and signaling molecules. Of these, many receptor-like kinases are implicated in cell wall damage signaling that have downstream effects on ROS signaling (143). Isoxaben was used to demonstrate that a receptor kinase, STRUBBELIG (SUB), mediates tissue morphogenesis in *Arabidopsis*, and signaling mediated by SUB has a regulatory effect on reduction of cellulose biosynthesis. This signaling can then affect the early increase of ROS after exposure to isoxaben when compared with the lack of increase in ROS accumulation in SUB-knockout mutants (138). THESEUS1, a receptor-like kinase involved in ROS production in response to isoxaben damage (139, 141), is required for ROS and lignin accumulation in roots (138). Similarly, isoxaben treatment was used to investigate how ROS and jasmonic acid, an important phytohormone involved in plant response to biotic and abiotic stress (144), are involved in lignin production after cell wall damage (139). This study reported that ROS production is necessary to induce a secondary ROS burst and jasmonic acid accumulation prior to inducing lignin deposition (139).

The *Arabidopsis* gene *AtRBOHD*, encoding an NADPH oxidase that catalyzes production of O_2_^•−^, is upregulated after treatment with isoxaben and thaxtomin A, another CBI herbicide (140). While thaxtomin A did not produce extracellular H_2_O_2_ in plants after treatment, this *AtRBOHD* gene was activated during plant defense and responsible for generating extracellular ROS (145). It was speculated that increased expression of this gene occurred to adjust the cell wall structure after it was affected by cellulose inhibition (140).

The precursor to ethylene, 1-aminocyclopropane-1-carboxylic acid (ACC), is involved in key regulation steps of the ethylene biosynthesis pathway. Regulation of ethylene production is important in plant development as ethylene is involved in many key stages of plant reproduction and germination. Tsang *et al.* (142) used CBIs to investigate a previously proposed idea that ACC can act as a signaling molecule independent of ethylene (146, 147). This was done by analyzing root elongation after treatment with isoxaben, dichlobenil, and thaxtomin A, another CBI. These studies confirmed that O_2_^•−^ production was required downstream of ACC biosynthesis.

Habituation treatments, or incremental increasing exposure over time, of CBIs influence the oxidative environment of plant cells. Maize cells under habituated dichlobenil treatment had reduced glutathione-*S*-transferase (GST), GR, and CAT activity, whereas there was no change in guaiacol-type peroxidase (GPOX) or APOX activity as compared with nonhabituated cells where GST, GR, CAT, GPOX, and APOX all increased in activity in the presence of dichlobenil (148). A study assessing the effects of habituation and dehabituation of dichlobenil on bean cells reported an increase in GPOX activity as compared with nonhabituated cells as well as a slightly higher increase in APOX activity in nonhabituated cells and then habituated and dehabituated cells (149). There was no change in CAT activity in any of the groups tested (149).

#### Group 30—inhibition of fatty acid thioesterase

The MoA of cinmethylin was recently assigned to fatty acid thioesterase (FAT), a new target site in fatty acid biosynthesis (150). FATs are plastid-localized enzymes that release fatty acids from their acyl carrier protein, as a step necessary to export fatty acids out of the chloroplast and transfer to the endoplasmic reticulum as fatty acyl-CoAs (151). While this herbicide causes loss of chlorophyll, this did not result in the formation of ROS, as measured by dihydroethidium staining (152). Dihydroethidium specifically reacts with O_2_^•−^ to form fluorescent 2-hydroxyethidium. Similar observations were made with the newer FAT inhibitor methiozolin (153).

#### Group 31—inhibition of serine–threonine protein phosphatases

Group 31 consists of a single herbicide, endothall. Endothall inhibits serine–threonine protein phosphatases (154). This class of enzymes are involved in the regulation of nearly every cellular process (*e.g.*, gene transcription and translation, metabolism, protein–protein interactions, protein activity, and apoptosis), and their inhibition leads to tissue desiccation and necrosis. Consequently, inhibition of serine–threonine protein phosphatases by endothall causes accumulation of ROS as a secondary effect of its impact on cell metabolism, and this can lead to an indirect loss of membrane integrity and necrosis (23, 155, 156). The browning of the treated tissue is likely associated with increased polyphenols because of loss of cellular compartmentation allows contact between polyphenols and polyphenol oxidase (155). In addition, desiccation and necrosis may be due to a secondary activity as an uncoupler at higher doses (156). See section on “Cell division and development” for more information on uncouplers.

### Other herbicide groups classified as inhibitors of cellular metabolism

The literature does not present any convincing evidence supporting the involvement of ROS in the injury caused by group 15 herbicides inhibiting very long-chain fatty acid elongases, group 18 herbicides inhibiting dihydropteroate synthase, or group 28 herbicides inhibiting dihydroorotate dehydrogenase.

## Cell division and development

**Figure 6:**
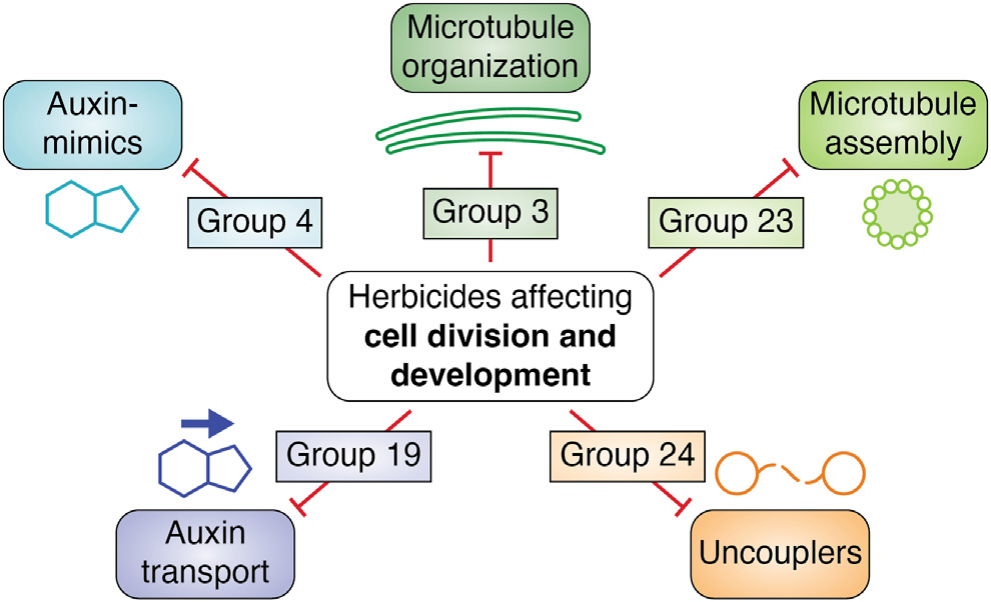
**Summary of HRAC groups affecting cell division and development.** The targets of herbicide groups affecting these processes are included. HRAC, Herbicide Resistance Action Committee.

This section covers herbicides classified by HRAC as inhibitors of cell division and development. These molecules target components of cellular growth through specific macromolecules and growth hormones (Fig. 6).

#### Group 4—auxin-mimic herbicides

Auxin-mimic herbicides are synthetic molecules that mimic the role of natural auxin hormones (*e.g.*, indole acetic acid) and disrupt most aspects of plant development, including nucleic acid metabolism and cell wall integrity. These synthetic auxins deregulate transcription, translation, and protein biosynthesis within the cell normally controlled by indole acetic acid, leading to unrestrained vascular growth, cell bursts, and cell and plant death (102).

Auxin herbicides and their interactions with ROS have been studied extensively as a secondary effect of their MoA. Of these herbicides, 2,4-dichlorophenoxyacetic acid (2,4-D) is one of the most widely used and most extensively researched. The main effect of 2,4-D is on the actin cytoskeleton by reducing actin bundling and polymerization (157), which leads to epinasty and impaired movement of mitochondria and peroxisomes, as they move along the cytoskeleton. As antioxidant organelles, impairment of the peroxisomes leads to reduced quenching of ROS and impairment of the mitochondria, as major cellular producers of ROS, can lead to an overproduction of ROS. The cell enters a state of severe oxidative stress because of limited placement of peroxisomes and mitochondrial function (158). Plant tissue death under 2,4-D is caused by the accumulation of abscisic acid and ethylene, inducing high ROS production and leading to a highly oxidizing environment. This leads to major changes in the cell wall by peroxidases and H_2_O_2_ cell wall reorganization (159). These ROS penetrate the plasma membrane leading to lipid peroxidation and loss of membrane integrity, electrolyte leakage of cytosol, and ultimately cell death (158).

Organ-specific oxidative stress from 2,4-D and other auxin-mimic herbicides is likely with no change in lipid peroxidation and increased antioxidant activities in stems of pea plants (160). In support of oxidative stress observed in leaves, Evan’s blue staining increased in *Azolla pinnata* plants treated with 2,4-D (161), correlating with an increase in oxidative stress. An increase in photosynthetic fluorescence of treated pea plant leaves was accompanied by H_2_O_2_ accumulation in mesophyll cells and secondary veins of *Arabidopsis thaliana* and O_2_^•−^ in veins and in mesophyll and epidermal cells (157, 162).

Antioxidative enzyme activity is an important indicator of a plant’s ability to handle oxidative stress, with increased activity indicating that a plant maintains an unimpaired antioxidative system. These antioxidant enzyme activity changes because of herbicides can also be organ specific as seen with increased acyl-CoA oxidase (ACOX) activity in foliar treatments of pea plants treated with 2,4-D but no observed difference in root treatments (163). Though McCarthy-Suarez *et al.* (164) reported that ACOX increased in both root and foliar treatments in pea plants under 2,4-D treated stress. A slight increase in CAT activity in root treatments of pea plants treated with 2,4-D, but no observed difference in foliar treatments have been reported. This is accompanied by an overall increase in SOD, GPOX, APOX, GR, GST, xanthine oxidase, glucose 6-phosphate dehydrogenase, and isocitrate dehydrogenase activity in both foliar and root treatments of pea plants and little to no observed change in glycolate oxidase and hydroxypyruvate reductase activity (163, 164, 165). These increases in antioxidative activity do not support the idea that auxin mimicry directly affects antioxidative machinery in plant cells. Glycolate oxidase activity decreased in young and mature leaves of the pea plant after 2,4-D treatment (162), suggesting a change in antioxidative machinery. On the other hand, GST, ACOX, SOD, xanthine oxidase, and lipoxygenase activity increased in treated leaves, whereas there was no change in CAT and GR activity.

As a result of 2,4-D treatment, the antioxidant enzyme machinery of plant cells remains mostly unaffected, while there is still accumulation of ROS produced by the proposed mechanism of the impaired actin cytoskeleton. Treatment of plants by 2,4-D increases intraperoxisomal H_2_O_2_ concentrations, and high accumulation was observed in the vascular tissues, central vein, and peripheral areas (157, 162, 163, 164, 165, 166). 2,4-D treatment also increased peroxisomal membrane O_2_^•−^ under foliar and root treatments of 2,4-D (164). Mutant plants for impaired ACOX 1 did not accumulate H_2_O_2_ (165), with ACOX suggested as a major source of ROS under 2,4-D (162). As a major indicator of oxidative stress, lipid peroxidation plays an important role in our understanding of oxidative stress in this herbicidal group. Treatment with 2,4-D on pea plants increased lipid peroxidation in foliar-treated plants (162, 163) and root treatments (164). In contrast, no increase in lipid peroxidation in pea plants under 2,4-D treatment was also found (165). Transcriptomic analyses also had an ROS-related peroxisomal footprint in early plant responses under 2,4-D treatment in wildtype *A. thaliana* plants (166).

Other auxin-mimic herbicides induce overproduction of ROS and/or increase oxidative stress after treatment, though the mechanisms are less studied than with 2,4-D. ROS accumulated in dicamba-, dichlorprop-, quinmerac-, and quinclorac-treated plants (167, 168, 169, 170). Increased O_2_^•−^ levels were also reported in dichlorprop- and quinclorac-treated plants (167, 170, 171), and increased OH^−^ levels were observed in quinclorac-treated plants (170). Chlorophyll content in *Echinochloa oryzicola* leaves decreased, and thiobarbituric acid reactive substance formation in shoots and ethane evolution, molecular indicators of lipid peroxidation, increased under quinclorac treatment in continuous light for 24 h (172). A significant increase in lipid peroxidation in roots and leaves of rice under quinclorac stress was observed (170).

#### Group 24—uncouplers

Herbicides that act as uncouplers of oxidative phosphorylation belong to the chemical family of dinitrophenols (*e.g.*, DNOC, dinosam, dinoseb, dinoterb, etinofen, medinoterb). They induce catastrophic ROS accumulation and subsequent lipid peroxidation that results in loss of membrane integrity (23, 173). Thus, they behave as contact herbicides that kill primarily annual weeds (174).

Uncouplers disrupt the normal coupling of respiratory electron transport and ATP synthesis in mitochondria and photosynthetic electron transport and photophosphorylation in chloroplasts. This results in a dissipation of the electrochemical proton gradients across the membranes of these organelles, resulting in the decrease of F-type ATP synthase activities while the electron transport chain continues to operate. Typically, uncouplers inhibit ATPase-driven phosphorylation at low concentrations and reduce electron transport chain at higher concentrations.

Mechanistically, uncoupling ATP synthesis from electron transport and proton translocation stimulated the electron flux through the electron transport chains and increased the formation of ROS. This effect is compounded by an overall decrease in the normal function of enzymes involved in ROS detoxification (175, 176, 177).

While there was an effort to discover new uncoupling herbicides in the early 1980s (178), these compounds often uncouple both chloroplastic photophosphorylation and respiratory oxidative phosphorylation in the mitochondria (179), and they tend to have broad toxicity (174). Therefore, the use of these compounds to manage weeds has been banned in many countries. Nonetheless, these compounds would be better classified if they were moved from the cell division and growth to the light activation of ROS category on the herbicide map.

### Other herbicide groups classified as inhibitors of cell division and development

The literature does not present any convincing evidence supporting the involvement of ROS in the injury caused by group 3 herbicides inhibiting microtubule assembly, group 19 herbicides inhibiting auxin transport, or group 23 herbicides inhibiting microtubule organization.

## Conclusion

Herbicides induce specific forms of stress to plants. In this review, we explore how that stress is expressed through ROS accumulation and alteration to the oxidative system in plant cells according to each HRAC MoA. As a group, herbicides in the light activation of ROS class are the main ones involved with ROS (Table 2). The HRAC classifies these herbicides as those with ROS-driven damage caused by light activation as a primary MoA. This is supported by the major MoAs in this group, but there is no evidence of ROS activity or oxidative stress in groups 12, 13, 27, 33, and 34. All the groups that directly inhibit photosynthesis involve high ROS production, that is, the main driver of damage in their activity. Group 14 herbicides disrupting porphyrin biosynthesis causes ROS accumulation in the cytosol and subsequent lipid peroxidation. Apart from group 32, most bleaching compounds do not exhibit ROS accumulation or any other evidence of oxidative stress as primary mechanisms or secondary mechanisms of damage in their activity. This seems at odds with their classification as light activation of ROS herbicides because they are light activated and no ROS accumulates. This pattern may warrant a reclassification of herbicides targeting carotenoid biosynthesis to the cellular metabolism category or an entirely new one. Though originally classified as a cellular metabolism MoA, glufosinate results in ROS overproduction because of downstream inhibition of photosynthetic carbon assimilation and photorespiration, and this accumulation acts as its primary mode of damage.

**Table 2:** Summary of HRAC herbicide modes of action classification 2022 and their association with ROS

Main group	Target site	Code	ROS^a^
Light activation of ROS			
	Photosynthesis at PSII—serine 264 binders	5	∗∗∗
	Photosynthesis at PSII—histidine 215 binders	6	∗∗∗
	Glutamine synthetase	10	∗∗∗
	PDS	12	
	Deoxy-d-xylulose phosphate synthase	13	
	Protoporphyrinogen oxidase	14	∗∗∗
	PSI electron diversion	22	∗∗∗
	Hydroxyphenylpyruvate dioxygenase	27	
	Solanesyl diphosphate synthase	32	
	HST	33	
	Lycopene cyclase	34	
Cellular metabolism			
	ACCase	1	∗
	ALS	2	∗
	EPSPS	9	∗
	Very long-chain fatty acid elongases	15	
	Dihydropteroate synthase	18	
	Dihydroorotate dehydrogenase	28	
	Cellulose synthesis^b^	29	∗
	FAT	30	
Cell division and development			
	Microtubule assembly	3	
	Auxin mimics	4	∗
	Auxin transport inhibitor	19	
	Microtubule organization	23	
	Uncouplers	24	∗∗∗
Others	Unknown^c^	0	

a Involvement of ROS in phytotoxic response. ROS accumulation as a primary mechanism (∗∗∗), secondary mechanism (∗∗), or indirectly (∗).

b Cellulose biosynthesis inhibitors used 29, 21, or 20 depending on the active ingredient in the old WSSA system. Now all in 29.

c Compounds with unknown MoA were in 11, 16, 17, 26, and 25 in the old WSSA system. Now they are all in 0.

Many of the herbicides involved in cellular metabolism induce some ROS production as a secondary consequence of their MoAs, often inflicting some oxidative damage, but secondary to their primary modes of damage. Though groups 15, 18, 28, and 30 are involved in cellular metabolism, there is no evidence in the literature that their MoA results in ROS accumulation or oxidative stress. A few herbicides involved in cell division and development also exhibit ROS production as a secondary consequence of their primary MoAs, notably auxin mimics and uncouplers. Most herbicides in the cell division and development section do not have any evidence in the literature of ROS accumulation or oxidative stress.

As we know, ROS production and accumulation is a natural consequence of stress. Some herbicide groups have no documented ROS accumulation, but that does not mean that there is no involvement of the oxidative system. Many of these groups are relatively new and understudied, so it is possible that further studies could find oxidative stress to be an aspect of their herbicidal activity, which could lead to a better understanding of their MoAs.

For example, a good understanding of the relationship between PQ and carotenoid biosynthesis has resulted in the development of synergistic mixtures of group 5 and 27 herbicides. Based on this review, additional synergistic relationships should be explored between group 5 herbicides and herbicide classes targeting other steps in PQ biosynthesis (*e.g.*, groups 32 and 33) (73, 74, 75). More recently, an unexpected biochemical crosstalk between glutamine and chlorophyll synthesis was discovered, resulting in ROS-driven synergism between group 10 and group 14 herbicides (180). A number of patents have recently been filed to protect this new use of herbicides (181). The role of ROS in other known synergistic and antagonistic interactions between herbicides should be further investigated (182).

## Conflict of interest

The authors declare that they have no conflicts of interest with the contents of this article.

## References

[1] Halliwell B. (2006). Reactive species and antioxidants. Redox biology is a fundamental theme of aerobic life. Plant Physiol..

[2] Foyer C.H., Noctor G. (2005). Redox homeostasis and antioxidant signaling: a metabolic interface between stress perception and physiological responses. Plant Cell.

[3] Szechyńska-Hebda M., Ghalami R.Z., Kamran M., Van Breusegem F., Karpiński S. (2022). To be or not to be? Are reactive oxygen species, antioxidants, and stress signalling universal determinants of life or death?. Cells.

[4] Foyer C.H. (2018). Reactive oxygen species, oxidative signaling and the regulation of photosynthesis. Environ. Exp. Bot..

[5] Apel K., Hirt H. (2004). Reactive oxygen species: metabolism, oxidative stress, and signal transduction. Annu. Rev. Plant Biol..

[6] Dmitrieva V.A., Tyutereva E.V., Voitsekhovskaja O.V. (2020). Singlet oxygen in plants: generation, detection, and signaling roles. Int. J. Mol. Sci..

[7] Gill S.S., Tuteja N. (2010). Reactive oxygen species and antioxidant machinery in abiotic stress tolerance in crop plants. Plant Physiol. Biochem..

[8] Juan C.A., Pérez de la Lastra J.M., Plou F.J., Pérez-Lebeña E. (2021). The chemistry of reactive oxygen species (ROS) revisited: outlining their role in biological macromolecules (DNA, lipids and proteins) and induced pathologies. Int. J. Mol. Sci..

[9] Watabe N., Ishida Y., Ochiai A., Tokuoka Y., Kawashima N. (2007). Oxidation decomposition of unsaturated fatty acids by singlet oxygen in phospholipid bilayer membranes. J. Oleo Sci..

[10] Noctor G., Foyer C.H. (2016). Intracellular redox compartmentation and ROS-related communication in regulation and signaling. Plant Physiol..

[11] D'Autréaux B., Toledano M.B. (2007). ROS as signalling molecules: mechanisms that generate specificity in ROS homeostasis. Nat. Rev. Mol. Cell Biol..

[12] Smirnoff N., Arnaud D. (2019). Hydrogen peroxide metabolism and functions in plants. New Phytol..

[13] Ransy C., Vaz C., Lombès A., Bouillaud F. (2020). Use of H_2_O_2_ to cause oxidative stress, the catalase issue. Int. J. Mol. Sci..

[14] del Río L.A., Corpas F.J., López-Huertas E., Palma J.M., Gupta D.K., Palma J.M., Corpas F.J. (2018). Plant Superoxide Dismutases: Function under Abiotic Stress Conditions in Antioxidants and Antioxidant Enzymes in Higher Plants.

[15] Demidchik V. (2015). Mechanisms of oxidative stress in plants: from classical chemistry to cell biology. Environ. Exp. Bot..

[16] Attri P., Kim Y.H., Park D.H., Park J.H., Hong Y.J., Uhm H.S. (2015). Generation mechanism of hydroxyl radical species and its lifetime prediction during the plasma-initiated ultraviolet (UV) photolysis. Sci. Rep..

[17] Demidchik V. (2017).

[18] Di Mascio P., Martinez G.R., Miyamoto S., Ronsein G.E., Medeiros M.H.G., Cadet J. (2019). Singlet molecular oxygen reactions with nucleic acids, lipids, and proteins. Chem. Rev..

[19] Driever S.M., Fryer M.J., Mullineaux P.M., Baker N.R., Pfannschmidt T. (2009). Imaging of Reactive Oxygen Species *in vivo* in Plant Signal Transduction: Methods and Protocols.

[20] Flors C., Fryer M.J., Waring J., Reeder B., Bechtold U., Mullineaux P.M. (2006). Imaging the production of singlet oxygen in vivo using a new fluorescent sensor, singlet oxygen sensor green. J. Exp. Bot..

[21] Caverzan A., Piasecki C., Chavarria G., Stewart C.N., Vargas L. (2019). Defenses against ROS in crops and weeds: the effects of interference and herbicides. Int. J. Mol. Sci..

[22] Bartoli C.G., Casalongué C.A., Simontacchi M., Marquez-Garcia B., Foyer C.H. (2013). Interactions between hormone and redox signalling pathways in the control of growth and cross tolerance to stress. Environ. Exp. Bot..

[23] Dayan F.E., Watson S.B. (2011). Plant cell membrane as a marker for light-dependent and light-independent herbicide mechanisms of action. Pestic. Biochem. Physiol..

[24] Krumova K., Cosa G., Nonell S., Flors C. (2016). Singlet Oxygen: Applications in Biosciences and Nanosciences.

[25] Kumar A., Prasad A., Sedlářová M., Pospíšil P. (2019). Organic radical imaging in plants: focus on protein radicals. Free Radic. Biol. Med..

[26] Dayan F.E., Duke S.O., Grossmann K. (2010). Herbicides as probes in plant biology. Weed Sci..

[27] Dayan F.E., Barker A., Takano H., Bough R., Ortiz M., Duke S.O., Grodzinski B. (2020). Herbicide Mechanisms of Action and Resistance in Comprehensive Biotechnology.

[28] Draber W., Tietjen K., Kluth J.F., Trebst A. (1991). Herbicides in photosynthesis research. Angew. Chem. Int. Ed..

[29] Tischer W., Strotmann H. (1977). Relationship between inhibitor binding by chloroplasts and inhibition of photosynthetic electron transport. Biochim. Biophysic Acta.

[30] Battaglino B., Grinzato A., Pagliano C. (2021). Binding properties of photosynthetic herbicides with the QB site of the D1 protein in plant photosystem II: a combined functional and molecular docking study. Plants (Basel).

[31] Broser M., Glöckner C., Gabdulkhakov A., Guskov A., Buchta J., Kern J. (2011). Structural basis of cyanobacterial photosystem II inhibition by the herbicide terbutryn. J. Biol. Chem..

[32] Lambreva M.D., Russo D., Polticelli F., Scognamiglio V., Antonacci A., Zobnina V. (2014). Structure/function/dynamics of photosystem II plastoquinone binding sites. Curr. Prot. Pept. Sci..

[33] Ido K., Gross C.M., Guerrero F., Sedoud A., Lai T.-L., Ifuku K. (2011). High and low potential forms of the Q_A_ quinone electron acceptor in Photosystem II of *Thermosynechococcus elongatus* and spinach. J. Photochem. Photobiol. B Biol..

[34] Pospíšil P. (2009). Production of reactive oxygen species by photosystem II. Biochim. Biophys. Acta.

[35] Fufezan C., Rutherford A.W., Krieger-Liszkay A. (2002). Singlet oxygen production in herbicide-treated photosystem II. FEBS Lett..

[36] Rutherford A.W., Krieger-Liszkay A. (2001). Herbicide-induced oxidative stress in photosystem II. Trends Biochem. Sci..

[37] Vavilin D.V., Vermaas W.F.J. (2000). Mutations in the CD-loop region of the D2 protein in *Synechocystis* sp. PCC 6803 modify charge recombination pathways in photosystem II *in vivo*. Biochemistry.

[38] Rappaport F., Guergova-Kuras M., Nixon P.J., Diner B.A., Lavergne J. (2002). Kinetics and pathways of charge recombination in photosystem II. Biochemistry.

[39] Akhavein A.A., Linscott D.L. (1968). The dipyridylium herbicides, paraquat and diquat. Residue Rev.

[40] Shen J.-R. (2015). The structure of photosystem II and the mechanism of water oxidation in photosynthesis. Annu. Rev. Plant Biol..

[41] Eberhard S., Finazzi G., Wollman F.-A. (2008). The dynamics of photosynthesis. Ann. Rev. Genet..

[42] Babbs C.F., Pham J.A., Coolbaugh R.C. (1989). Lethal hydroxyl radical production in paraquat-treated plants. Plant Physiol..

[43] Hess F.D. (2000). Light-dependent herbicides: an overview. Weed Sci..

[44] Mehler A.H. (1951). Studies on reactions of illuminated chloroplasts: I. Mechanism of the reduction of oxygen and other hill reagents. Arch. Biochem. Biophys..

[45] Mehler A.H. (1951). Studies on reactions of illuminated chloroplasts. II. Stimulation and inhibition of the reaction with molecular oxygen. Arch. Biochem. Biophys..

[46] Noctor G., Foyer C.H. (1998). Ascorbate and glutathione: keeping active oxygen under control. Annu. Rev. Plant Physiol. Plant Mol. Biol..

[47] Hippeli S., Heiser I., Elstner E.F. (1999). Activated oxygen and free oxygen radicals in pathology: new insights and analogies between animals and plants. Plant Physiol. Biochem..

[48] Saran M., Michel C., Bors W. (1998). Radical functions *in vivo*: a critical review of current concepts and hypotheses. Z. Naturforsch. C J. Biosci..

[49] Johnson W.O., Kollman G.E., Swithenbank C., Yih R.Y. (1978). RH-6201 (Blazer): a new broad spectrum herbicide for postemergence use in soybean. J. Agric. Food Chem..

[50] Bryant D.A., Hunter C.N., Warren M.J. (2020). Biosynthesis of the modified tetrapyrroles—the pigments of life. J. Biol. Chem..

[51] Dayan F.E., Dayan E. (2011). Porphyrins: one ring in the colors of life. Am. Sci..

[52] Dayan F.E., Duke S.O., Krieger R., Doull J., Hodgson E., Maibach H., Reiter L., Ritter L. (2010). Protoporphyrinogen Oxidase-Inhibiting Herbicides in Haye's Handbook of Pesticide Toxicology.

[53] Becerril J.M., Duke S.O. (1989). Protoporphyrin IX content correlates with activity of photobleaching herbicides. Plant Physiol..

[54] Lee H.J., Duke S.O. (1994). Protoporphyrinogen IX-oxidizing activities involved in the mode of action of peroxidizing herbicides. J. Agric. Food Chem..

[55] Lee J.J., Matsumoto H., Ishizuka K. (1992). Light involvement in oxyfluorfen-induced protoporphyrin IX accumulation in several species of intact plants. Pestic. Biochem. Physiol..

[56] Matringe M., Camadro J.-M., Labbe P., Scalla R. (1989). Protoporphyrinogen oxidase as a molecular target for diphenyl ether herbicides. Biochem. J..

[57] Dayan F.E., Weete J.D., Duke S.O., Hancock H.G. (1997). Soybean (*Glycine max*) cultivar differences in response to sulfentrazone. Weed Sci..

[58] Dayan F.E., Duke S.O., Weete J.D., Hancock H.G. (1997). Selectivity and mode of action of carfentrazone-ethyl, a novel phenyl triazolinone herbicide. Pestic. Sci..

[59] Duke S.O., Kenyon W.H., Böger P., Sandmann G. (1993). Target Assays for Modern Herbicides and Related Compounds.

[60] Nandihalli U.B., Sherman T.D., Duke M.V., Fisher J.D., Musco V.A., Becerril J.M. (1992). Correlation of protoporphyrinogen oxidase inhibition by *O*-phenyl pyrrolidino- and piperidino-carbamates with their herbicidal effects. Pestic. Sci..

[61] Kenyon W.H., Duke S.O., Vaughn K.C. (1985). Sequences of effects of acifluorfen on physiological and ultrastructural parameters in cucumber cotyledon discs. Pestic. Biochem. Physiol..

[62] Tripathy B.C., Mohapatra A., Gupta I. (2007). Impairment of the photosynthetic apparatus by oxidative stress induced by photosensitization reaction of protoporphyrin IX. Biochim. Biophys. Acta.

[63] Dayan F.E., Barker A., Dayan L.C., Ravet K. (2019). The role of antioxidants in the protection of plants against inhibitors of protoporphyrinogen oxidase. React. Oxygen Species.

[64] Trebst A., Depka B. (1997). Role of carotene in the rapid turnover and assembly of photosystem II in *Chlamydomonas reinhardtii*. FEBS Lett..

[65] Sandmann G., Ward C.E., Lo W.C., Nagy J.O., Böger P. (1990). Bleaching herbicide flurtamone interferes with phytoene desaturase. Plant Physiol..

[66] Fedtke C., Depka B., Schallner O., Tietjen K., Trebst A., Wollweber D. (2001). Mode of action of new diethylamines in lycopene cyclase inhibition and in photosystem II turnover. Pest Manag. Sci..

[67] Armel G.R., Rardon P.L., McComrick M.C., Ferry N.M. (2007). Differential response of several carotenoid biosynthesis inhibitors in mixtures with atrazine. Weed Technol..

[68] Sandmann G., Schmidt A., Linden H., Böger P. (1991). Phytoene desaturase, the essential target for bleaching herbicides. Weed Sci..

[69] Chang H.-L., Hsu Y.-T., Kang C.-Y., Lee T.-M. (2013). Nitric oxide down-regulation of carotenoid synthesis and PSII activity in relation to very high light-induced singlet oxygen production and oxidative stress in *Chlamydomonas reinhardtii*. Plant Cell Physiol..

[70] Jung S., Kim J.S., Cho K.Y., Tae G.S., Kang B.G. (2000). Antioxidant responses of cucumber (*Cucumis sativus*) to photoinhibition and oxidative stress induced by norflurazon under high and low PPFDs. Plant Sci..

[71] Darwish M., Vidal V., Lopez-Lauri F., Alnaser O., Junglee S., El Maataoui M. (2015). Tolerance to clomazone herbicide is linked to the state of LHC, PQ-pool and ROS detoxification in tobacco (*Nicotiana tabacum* L.). J. Plant Physiol..

[72] Norris S.R., Barrette T.R., DellaPenna D. (1995). Genetic dissection of carotenoid synthesis in *Arabidopsis* defines plastoquinone as an essential component of phytoene desaturation. Plant Cell.

[73] Abendroth J.A., Martin A.R., Roeth F.W. (2006). Plant response to combinations of mesotrione and photosystem II inhibitors. Weed Technol..

[74] Armel G.R., Hall G.J., Wilson H.P., Cullen N. (2005). Mesotrione plus atrazine mixtures for control of Canada thistle (*Cirsium arvense*). Weed Sci..

[75] Johnson B.C., Young B.G., Matthews J.L. (2002). Effect of postemergence application rate and timing of mesotrione on corn (*Zea mays*) response and weed control. Weed Technol..

[76] Kahlau S., Schröder F., Freigang J., Laber B., Lange G., Passon D. (2020). Aclonifen targets solanesyl diphosphate synthase, representing a novel mode of action for herbicides. Pest Manag. Sci..

[77] Kilinc Ö., Reynaud S., Perez L., Tissut M., Ravanel P. (2009). Physiological and biochemical modes of action of the diphenylether aclonifen. Pestic. Biochem. Physiol..

[78] Sadre R., Frentzen M., Saeed M., Hawkes T. (2010). Catalytic reactions of the homogentisate prenyl transferase involved in plastoquinone-9 biosynthesis. J. Biol. Chem..

[79] Shino M., Hamada T., Shigematsu Y., Hirase K., Banba S. (2018). Action mechanism of bleaching herbicide cyclopyrimorate, a novel homogentisate solanesyltransferase inhibitor. J. Pestic. Sci..

[80] Shino M., Hamada T., Shigematsu Y., Banba S. (2020). *In vivo* and *in vitro* evidence for the inhibition of homogentisate solanesyltransferase by cyclopyrimorate. Pest Manag. Sci..

[81] Castelfranco P., Brown M.S. (1963). A hypothesis of amitrole action based on its behavior toward free radical generating systems. Weeds.

[82] Hilton J.L., Kearney P.C., Ames B.N. (1965). Mode of action of the herbicide, 3-amino-1,2,4-triazole (amitrole): inhibition of an enzyme of histidine biosynthesis. Arch. Biochem. Biophys..

[83] Saidak W.J., Marriage P.B. (1976). Response of Canada thistle varieties to amitrole and glyphosate. Can. J. Plant Sci..

[84] Burns E.R., Buchanan G.A., Carter M.C. (1971). Inhibition of carotenoid synthesis as a mechanism of action of amitrole, dichlormate, and pyriclor. Plant Physiol..

[85] Agnolucci L., Vecchia F.D., Barbato R., Tassani V., Casadoro G., Rascio N. (1996). Amitrole effects on chloroplasts of barley plants grown at different temperatures. J. Plant Physiol..

[86] Rascio N., Vecchia F.D., Agnolucci L., Barbato R., Tassani V., Casadoro G. (1996). Amitrole effects on barley etioplasts. J. Plant Physiol..

[87] Heim D.R., Larrinua I.M. (1989). Primary site of action of amitrole in *Arabidopsis thaliana* involves inhibition of root elongation but not of histidine or pigment biosynthesis. Plant Physiol..

[88] Bouvier F., d'harlingue A., Camara B. (1997). Molecular analysis of carotenoid cyclase inhibition. Arch. Biochem. Biophys..

[89] Hoerlein G., Ware G.W. (1994). Glufosinate (Phosphinothricin), a Natural Amino Acid with Unexpected Herbicidal Properties in Reviews of Environmental Contamination and Toxicology: Continuation of Residue Reviews.

[90] Lea P.J., Miflin B.J. (2011). Nitrogen assimilation and its relevance to crop improvement. Ann. Plant Rev..

[91] Bernard S.M., Habash D.Z. (2009). The importance of cytosolic glutamine synthetase in nitrogen assimilation and recycling. New Phytol..

[92] Manderscheid R., Böger P., Sandmann G. (1993). Irreversible Inhibition of Glutamine Synthetase from Higher Plants by the Herbicide Phosphinothricin in Target Assays for Modern Herbicides and Related Phytotoxic Compounds.

[93] Wild A., Sauer H., Ruhle W. (1987). The effect of phosphinothricin (glufosinate) on photosynthesis. I. Inhibition of photosynthesis and accumulation of ammonia. Z. Naturforsch. C J. Biosci..

[94] Wendler C., Barniske M., Wild A. (1990). Effect of phosphinothricin (glufosinate) on photosynthesis and photorespiration of C_3_ and C_4_ plants. Photosynth. Res..

[95] Wild A., Wendler C. (1993). Inhibitory action of glufosinate on photosynthesis. Z. Naturforsch. C J. Biosci..

[96] Wendler C., Putzer A., Wild A. (1992). Effect of glufosinate (phosphinothricin) and inhibitors of photorespiration on photosynthesis and ribulose-1,5-bisphosphate carboxylase activity. J. Plant Physiol..

[97] Coetzer E., Al-Khatib K. (2001). Photosynthetic inhibition and ammonium accumulation in Palmer amaranth after glufosinate application. Weed Sci..

[98] Takano H.K., Beffa R., Preston C., Westra P., Dayan F.E. (2019). Reactive oxygen species trigger the fast action of glufosinate. Planta.

[99] Takano H.K., Beffa R., Preston C., Westra P., Dayan F.E. (2020). A novel insight into the mode of action of glufosinate: how reactive oxygen species are formed. Photosynth. Res..

[100] Lu Y., Li Y., Yang Q., Zhang Z., Chen Y., Zhang S. (2014). Suppression of glycolate oxidase causes glyoxylate accumulation that inhibits photosynthesis through deactivating Rubisco in rice. Physiol. Plant.

[101] Cobb A.H., Reade J.P.H. (2010).

[102] Sherwani S.I., Arif I.A., Khan H.A. (2015). Herbicides, Physiology of Action, and Safety.

[103] Radwan D.E.M. (2012). Salicylic acid induced alleviation of oxidative stress caused by clethodim in maize (*Zea mays* L.) leaves. Pestic. Biochem. Physiol..

[104] Fayez K.A., Radwan D.E.M., Mohamed A.K., Abdelrahman A.M. (2014). Fusilade herbicide causes alterations in chloroplast ultrastructure, pigment content and physiological activities of peanut leaves. Photosynthetica.

[105] Luo X.-Y., Sunohara Y., Matsumoto H. (2004). Fluazifop-butyl causes membrane peroxidation in the herbicide-susceptible broad leaf weed bristly starbur (*Acanthospermum hispidum*). Pestic. Biochem. Physiol..

[106] Durner J., Gailus V., Böger P. (1994). The oxygenase reaction of acetolactate synthase detected by chemiluminescence. FEBS Lett..

[107] Zabalza A., Gaston S., Sandalio L.M., del Río L.A., Royuela M. (2007). Oxidative stress is not related to the mode of action of herbicides that inhibit acetolactate synthase. Environ. Exp. Bot..

[108] Agostinetto D., Perboni L.T., Langaro A.C., Gomes J., Fraga D.S., Franco J.J. (2016). Changes in photosynthesis and oxidative stress in wheat plants submmited to herbicides application. Planta Daninha.

[109] Hassan N.M., Nemat Alla M.M. (2005). Oxidative stress in herbicide-treated broad bean and maize plants. Acta Physiol. Plant..

[110] Nohatto M.A., Agostinetto D., Langaro A.C., de Oliveira C., Ruchel Q. (2016). Antioxidant activity of rice plants sprayed with herbicides. Pesquisa Agropecuária Trop..

[111] Qian H., Lu T., Peng X., Han X., Fu Z., Liu W. (2011). Enantioselective phytotoxicity of the herbicide imazethapyr on the response of the antioxidant system and starch metabolism in *Arabidopsis thaliana*. PloS one.

[112] Liu X.-Q., Yu C.-Y., Dong J.-G., Hu S.-W., Xu A.-X. (2017). Acetolactate synthase-inhibiting gametocide amidosulfuron causes chloroplast destruction, tissue autophagy, and elevation of ethylene release in rapeseed. Front. Plant Sci..

[113] Sun C., Chen S., Jin Y., Song H., Ruan S., Fu Z. (2016). Effects of the herbicide imazethapyr on photosynthesis in PGR5- and NDH-deficient *Arabidopsis thaliana* at the biochemical, transcriptomic, and proteomic levels. J. Agric. Food Chem..

[114] Gomes M.P., da Silva Cruz F.V., Bicalho E.M., Borges F.V., Fonseca M.B., Juneau P. (2017). Effects of glyphosate acid and the glyphosate-commercial formulation (Roundup) on *Dimorphandra wilsonii* seed germination: interference of seed respiratory metabolism. Environ. Pollut..

[115] Ahsan N., Lee D.-G., Lee K.-W., Alam I., Lee S.-H., Bahk J.D. (2008). Glyphosate-induced oxidative stress in rice leaves revealed by proteomic approach. Plant Physiol. Biochem..

[116] Camilo dos Santos J.C., Ribeiro Silva D.M., Jardim Amorim D., do Rosário Rosa V., Farias dos Santos A.L., Domingues Velini E. (2022). Glyphosate hormesis attenuates water deficit stress in safflower (*Carthamus tinctorius* L.) by modulating physiological and biochemical mediators. Sci. Total Environ..

[117] Cruz C.E.S., de Freitas-Silva L., Ribeiro C., da Silva L.C. (2021). Physiological and morphoanatomical effects of glyphosate in *Eugenia uniflora*, a Brazilian plant species native to the Atlantic Forest biome. Environ. Sci. Pollut. Res..

[118] de Freitas-Silva L., Rodríguez-Ruiz M., Houmani H., da Silva L.C., Palma J.M., Corpas F.J. (2017). Glyphosate-induced oxidative stress in *Arabidopsis thaliana* affecting peroxisomal metabolism and triggers activity in the oxidative phase of the pentose phosphate pathway (OxPPP) involved in NADPH generation. J. Plant Physiol..

[119] Gomes M.P., Juneau P. (2016). Oxidative stress in duckweed (*Lemna minor* L.) induced by glyphosate: is the mitochondrial electron transport chain a target of this herbicide?. Environ. Pollut..

[120] Gomes M.P., Le Manac'h S.G., Maccario S., Labrecque M., Lucotte M., Juneau P. (2016). Differential effects of glyphosate and aminomethylphosphonic acid (AMPA) on photosynthesis and chlorophyll metabolism in willow plants. Pestic. Biochem. Physiol..

[121] Gomes M.P., Le Manac’h S.G., Hénault-Ethier L., Labrecque M., Lucotte M., Juneau P. (2017). Glyphosate-dependent inhibition of photosynthesis in willow. Front. Plant Sci..

[122] Gomes M.P., Le Manac’h S.G., Moingt M., Smedbol E., Paquet S., Labrecque M. (2016). Impact of phosphate on glyphosate uptake and toxicity in willow. J. Hazard. Mater..

[123] Gomes M.P., Smedbol E., Chalifour A., Hénault-Ethier L., Labrecque M., Lepage L. (2014). Alteration of plant physiology by glyphosate and its by-product aminomethylphosphonic acid: an overview. J. Exp. Bot..

[124] Ke M., Ye Y., Zhang Z., Gillings M., Qu Q., Xu N. (2021). Synergistic effects of glyphosate and multiwall carbon nanotubes on *Arabidopsis thaliana* physiology and metabolism. Sci. Total Environ..

[125] Miteva L.P.E., Ivanov S.V., Alexieva V.S. (2010). Alterations in glutathione pool and some related enzymes in leaves and roots of pea plants treated with the herbicide glyphosate. Russ. J. Plant Physiol..

[126] Percival G.C. (2017). The influence of glyphosate on carotenoid pigments, reactive oxygen species scavenging enzymes and secondary stress metabolites within leaf tissue of three Acer species. Urban For. Urban Green..

[127] Radwan D.E.M., Fayez K.A. (2016). Photosynthesis, antioxidant status and gas-exchange are altered by glyphosate application in peanut leaves. Photosynthetica.

[128] Shopova E., Katerova Z., Brankova L., Dimitrova L., Sergiev I., Todorova D. (2021). Modulation of physiological stress response of *Triticum aestivum* L. to glyphosate by brassinosteroid application. Life.

[129] Singh H., Singh N.B., Singh A., Hussain I., Yadav V. (2017). Physiological and biochemical roles of nitric oxide against toxicity produced by glyphosate herbicide in *Pisum sativum*. Russ. J. Plant Physiol..

[130] Sergiev I.G., Alexieva V.S., Ivanov S.V., Moskova I.I., Karanov E.N. (2006). The phenylurea cytokinin 4PU-30 protects maize plants against glyphosate action. Pestic. Biochem. Physiol..

[131] Gomes M.P., Bicalho E.M., Smedbol É., Cruz F.V., Lucotte M., Garcia Q.S. (2017). Glyphosate can decrease germination of glyphosate-resistant soybeans. J. Agric. Food Chem..

[132] Moretti M.L., Van Horn C.R., Robertson R., Segobye K., Weller S.C., Young B.G. (2018). Glyphosate resistance in *Ambrosia trifida*: Part 2. Rapid response physiology and non-target-site resistance. Pest Manag. Sci..

[133] Harre N.T., Nie H., Jiang Y., Young B.G. (2018). Differential antioxidant enzyme activity in rapid-response glyphosate-resistant *Ambrosia trifida*. Pest Manag. Sci..

[134] Piasecki C., Carvalho I.R., Avila L.A., Agostinetto D., Vargas L. (2020). Glyphosate and saflufenacil: Elucidating their combined action on the control of glyphosate-resistant *Conyza bonariensis*. Agriculture.

[135] Eceiza M.V., Gil-Monreal M., Barco-Antoñanzas M., Zabalza A., Royuela M. (2022). The moderate oxidative stress induced by glyphosate is not detected in *Amaranthus palmeri* plants overexpressing EPSPS. J. Plant Physiol..

[136] Maroli A.S., Nandula V.K., Dayan F.E., Duke S.O., Gerard P., Tharayil N. (2015). Metabolic profiling and enzyme analyses indicate a potential role of antioxidant systems in complementing glyphosate resistance in an *Amaranthus palmeri* biotype. J. Agric. Food Chem..

[137] Passardi F., Penel C., Dunand C. (2004). Performing the paradoxical: how plant peroxidases modify the cell wall. Trends Plant Sci..

[138] Chaudhary A., Chen X., Gao J., Leśniewska B., Hammerl R., Dawid C. (2020). The Arabidopsis receptor kinase STRUBBELIG regulates the response to cellulose deficiency. PLoS Genet..

[139] Denness L., McKenna J.F., Segonzac C., Wormit A., Madhou P., Bennett M. (2011). Cell wall damage-induced lignin biosynthesis is regulated by a reactive oxygen species- and jasmonic acid-dependent process in *Arabidopsis*. Plant Physiol..

[140] Duval I., Beaudoin N. (2009). Transcriptional profiling in response to inhibition of cellulose synthesis by thaxtomin A and isoxaben in *Arabidopsis thaliana* suspension cells. Plant Cell Rep..

[141] Hamann T., Bennett M., Mansfield J., Somerville C. (2009). Identification of cell-wall stress as a hexose-dependent and osmosensitive regulator of plant responses. Plant J..

[142] Tsang D.L., Edmond C., Harrington J.L., Nühse T.S. (2011). Cell wall integrity controls root elongation via a general 1-aminocyclopropane-1-carboxylic acid-dependent, ethylene-independent pathway. Plant Physiol..

[143] Engelsdorf T., Hamann T. (2014). An update on receptor-like kinase involvement in the maintenance of plant cell wall integrity. Ann. Bot..

[144] Ruan J., Zhou Y., Zhou M., Yan J., Khurshid M., Weng W. (2019). Jasmonic acid signaling pathway in plants. Int. J. Mol. Sci..

[145] Torres M.A., Dangl J.L., Jones J.D.G. (2002). *Arabidopsis* gp91^phox^ homologues *AtrbohD* and *AtrbohF* are required for accumulation of reactive oxygen intermediates in the plant defense response. Proc. Natl. Acad. Sci. U. S. A..

[146] Tsuchisaka A., Yu G., Jin H., Alonso J.M., Ecker J.R., Zhang X. (2009). A combinatorial interplay among the 1-aminocyclopropane-1-carboxylate isoforms regulates ethylene biosynthesis in *Arabidopsis thaliana*. Genetics.

[147] Xu S.L., Rahman A., Baskin T.I., Kieber J.J. (2008). Two leucine-rich repeat receptor kinases mediate signaling, linking cell wall biosynthesis and ACC synthase in *Arabidopsis*. Plant Cell.

[148] Mélida H., Encina A., Álvarez J., Acebes J.L., Caparrós-Ruiz D. (2010). Unraveling the biochemical and molecular networks involved in maize cell habituation to the cellulose biosynthesis inhibitor dichlobenil. Mol. Plant.

[149] García-Angulo P., Alonso-Simón A., Mélida H., Encina A., Acebes J.L., Álvarez J.M. (2009). High peroxidase activity and stable changes in the cell wall are related to dichlobenil tolerance. J. Plant Physiol..

[150] Campe R., Hollenbach E., Kämmerer L., Hendriks J., Höffken H.W., Kraus H. (2018). A new herbicidal site of action: cinmethylin binds to acyl-ACP thioesterase and inhibits plant fatty acid biosynthesis. Pestic. Biochem. Physiol..

[151] Dayan F.E. (2019). Current status and future prospects in herbicide discovery. Plants (Basel).

[152] Grossmann K., Hutzler J., Tresch S., Christiansen N., Looser R., Ehrhardt T. (2012). On the mode of action of the herbicides cinmethylin and 5-benzyloxymethyl-1,2-isoxazolines: putative inhibitors of plant tyrosine aminotransferase. Pest Manag. Sci..

[153] Brabham C., Johnen P., Hendriks J., Betz M., Zimmermann A., Gollihue J. (2021). Herbicide symptomology and the mechanism of action of methiozolin. Weed Sci..

[154] Bajsa J., Pan Z., Dayan F.E., Owens D.K., Duke S.O. (2012). Validation of serine-threonine protein phosphatase as the herbicide target site of endothall. Pestic. Biochem. Physiol..

[155] Rikin A., Rubin B. (1983). Increase of cotton cotyledon resistance to the herbicide endothall by abscisic acid. Physiol. Plant.

[156] Tresch S., Schmotz J., Grossmann K. (2011). Probing mode of action in plant cell cycle by the herbicide endothall, a protein phosphatase inhibitor. Pestic. Biochem. Physiol..

[157] Rodríguez-Serrano M., Pazmiño D.M., Sparkes I., Rochetti A., Hawes C., Romero-Puertas M.C. (2014). 2,4-Dichlorophenoxyacetic acid promotes S-nitrosylation and oxidation of actin affecting cytoskeleton and peroxisomal dynamics. J. Exp. Bot..

[158] Christoffoleti P.J., de Figueiredo M.R.A., Peres L.E.P., Nissen S., Gaines T. (2015). Auxinic herbicides, mechanisms of action, and weed resistance: a look into recent plant science advances. Sci. Agric..

[159] Pereira C.S., Ribeiro J.M., Vatulescu A.D., Findlay K., MacDougall A.J., Jackson P.A. (2011). Extensin network formation in *Vitis vinifera* callus cells is an essential and causal event in rapid and H_2_O_2_-induced reduction in primary cell wall hydration. BMC Plant Biol..

[160] McCarthy-Suárez I., Gómez M., del Río L.A., Palma J.M. (2011). Organ-specific effects of the auxin herbicide 2,4-D on the oxidative stress and senescence-related parameters of the stems of pea plants. Acta Physiol. Plant..

[161] De A., Dey N., Adak M. (2016). Some physiological insights of 2,4-D sensitivity in an aquatic fern: *Azolla pinnata* R.Br.. J. Biotechnol. Biomater..

[162] Pazmiño D.M., Rodriguez-Serrano M., Romero-Puertas M.C., Archilla-Ruiz A., Del Rio L.A., Sandalio L.M. (2011). Differential response of young and adult leaves to herbicide 2,4-dichlorophenoxyacetic acid in pea plants: role of reactive oxygen species. Plant Cell Environ..

[163] Romero-Puertas M.C., McCarthy I., Gomez M., Sandalio L.M., Corpas F.J., Del Rio L.A. (2004). Reactive oxygen species-mediated enzymatic systems envolved in the oxidative action of 2,4-dichlorophenoxyacetic acid. Plant Cell Environ..

[164] McCarthy-Suárez I., Gómez M., Del Río L.A., Palma J.M. (2011). Role of peroxisomes in the oxidative injury induced by 2,4-dichlorophenoxyacetic acid in leaves of pea plants. Biol. Plantarum.

[165] Todorova D., Sergiev I., Shopova E., Brankova L., Jankauskienė J., Jurkonienė S. (2021). Physiological responses of pea plants to treatment with synthetic auxins and auxin-type herbicide. Botanica.

[166] Romero-Puertas M.C., Peláez-Vico M.Á., Pazmiño D.M., Rodríguez-Serrano M., Terrón-Camero L., Bautista R. (2022). Insights into ROS-dependent signalling underlying transcriptomic plant responses to the herbicide 2,4-D. Plant Cell Environ..

[167] Chen H., Qin Y., Pu J., Hu J., Wen Y. (2021). Phytotoxicity of the chiral herbicide dichlorprop: cross-talk between nitric oxide, reactive oxygen species and phytohormones. Sci. Total Environ..

[168] Grossmann K., Kwiatkowski J., Tresch S. (2001). Auxin herbicides induce H_2_O_2_ overproduction and tissue damage in cleavers (*Galium aparine* L.). J. Exp. Bot..

[169] Johnston C.R., Vencill W.K., Grey T.L., Stanley Culpepper A., Henry G.M., Czarnota M.A. (2019). Investigation into interactions of environmental and application time effects on 2,4-D and dicamba-induced phytotoxicity and hydrogen peroxide formation. Weed Sci..

[170] Wang J., Lv M., Islam F., Gill R.A., Yang C., Ali B. (2016). Salicylic acid mediates antioxidant defense system and ABA pathway related gene expression in *Oryza sativa* against quinclorac toxicity. Ecotoxicol. Environ. Saf..

[171] Sunohara Y., Matsumoto H. (2008). Quinclorac-induced cell death is accompanied by generation of reactive oxygen species in maize root tissue. Phytochemistry.

[172] Sunohara Y., Matsumoto H. (2004). Oxidative injury induced by the herbicide quinclorac on *Echinochloa oryzicola* Vasing. and the involvement of antioxidative ability in its highly selective action in grass species. Plant Sci..

[173] Halim N.A., Ma N.L., Sahid I., Chuah T.S. (2018). Effects of 2, 4-di-tert-butylphenol and selected herbicides which induced lipid peroxidation on quantum yield and membrane integrity of weedy plants under dark and light conditions. Sains Malays..

[174] Jurado A., Fernandes M.A.S., Videira R., Peixoto F.P., Vicente J.A.F., Kortekamp A. (2011). Herbicides *and Environment*.

[175] Cadenas E., Davies K.J.A. (2000). Mitochondrial free radical generation, oxidative stress, and aging. Free Radic. Biol. Med..

[176] Lambert A.J., Brand M.D. (2004). Superoxide production by NADH:ubiquinone oxidoreductase (complex I) depends on the pH gradient across the mitochondrial inner membrane. Biochem. J..

[177] Aranha M.M., Matos A.R., Teresa Mendes A., Vaz Pinto V., Rodrigues C.M.P., Arrabaça J.D. (2007). Dinitro-o-cresol induces apoptosis-like cell death but not alternative oxidase expression in soybean cells. J. Plant Physiol..

[178] Wright B.J., Baillie A.C., Wright K., Dowsett J.R., Sharpe T.M. (1980). Synthesis of potential herbicides designed to uncouple photophosphorylation. Phytochemistry.

[179] Moreland D.E. (1993). Research on biochemistry of herbicides: an historical overview. Z. Naturforsch. C.

[180] Takano H.K., Beffa R., Preston C., Westra P., Dayan F.E. (2020). Glufosinate enhances the activity of protoporphyrinogen oxidase inhibitors. Weed Sci..

[181] Dayan F., Takano H., Westra P., Bowe S., Liebl R.A., Findley D. (2023).

[182] Barbieri G.F., Young B.G., Dayan F.E., Streibig J.C., Takano H., Merotto A.J. (2022). Herbicide mixtures: interactions and modeling. Adv. Weed Sci..

[183] Imlay J.A. (2003). Pathways of oxidative damage. Ann. Rev. Microbiol..

